# DNA Methylation Signatures of Cellular Senescence Are Not Reversed by Senolytic Treatment

**DOI:** 10.1111/acel.70430

**Published:** 2026-02-26

**Authors:** Jessica Kasamoto, John González, Yaroslav Markov, Raghav Sehgal, Edwin Lee, Varun B. Dwaraka, Ryan Smith, Albert T. Higgins‐Chen

**Affiliations:** ^1^ Program in Computational Biology and Bioinformatics Yale University New Haven Connecticut USA; ^2^ Department of Pathology Yale University School of Medicine New Haven Connecticut USA; ^3^ Department of Psychiatry Yale University School of Medicine New Haven Connecticut USA; ^4^ Institute for Hormonal Balance Orlando Florida USA; ^5^ TruDiagnostic Lexington Kentucky USA

**Keywords:** aging, biomarker, epigenetic clock, senescence, senolytics

## Abstract

Epigenetic clocks are commonly used aging biomarkers based on DNA methylation that predict long‐term morbidity and mortality risk. Increased cellular senescence with age is also posited to contribute to age‐related disease and mortality. However, prior studies have found that existing epigenetic clocks show inconsistent associations with cellular senescence and no reductions after senolytic treatment. We hypothesize this reflects that senescence‐related CpGs are a small proportion of age‐related CpGs, and that an epigenetic clock focused on a core senescence signal conserved across different cell types and different senescence inducers would be a better tool for monitoring senescence and senolytic treatment compared to traditional epigenetic clocks. In our study, we find that senescence, age and mortality risk intersect at a small subset of the DNA methylome (9363 CpGs out of 396,333 analyzed; 2.4%). Utilizing these CpGs, we generated three different epigenetic clocks trained to predict in vitro senescence, age, and mortality, respectively. Surprisingly, all three of these predictors stayed the same or even accelerated after senolytic treatment in both in vivo and in vitro data. Our findings not only call into question whether cellular senescence can be captured by DNA methylation but also challenge the assumption that aging biomarkers decrease after geroscience interventions.

## Graphical abstract

We found very little overlap between CpGs that were correlated with in vitro senescence, chronological age, and mortality. While we were able to train epigenetic clocks with CpGs that accelerated with cellular senescence, these clocks did not decelerate with Senolytic treatment.

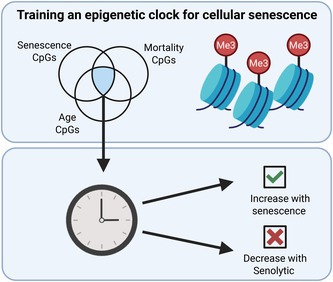

## 1 Introduction

According to the geroscience hypothesis, therapeutic strategies that alter the pace of aging have large potential in the field of preventative medicine, allowing us to delay the onset or severity of age‐related diseases (Hornsby 2021; Justice et al. 2018). Biological age uses biophysiological measurements to determine an individual's age‐related risk of negative health outcomes. Epigenetic clocks are one type of biological age algorithm based on DNA methylation (DNAm). Many studies have shown that increased biological age, according to epigenetic clocks, corresponds to an elevated risk of age‐related disease and mortality (Chervova et al. 2024). A major question is whether epigenetic clocks could also be early indicators of the efficacy of anti‐aging treatments, measurable on the time scale of clinical trials (Moqri et al. 2023).

Cellular senescence is a well‐studied driver of age‐related disease that can be targeted pharmacologically, and aging biomarkers that could monitor both the progression and reversal of cellular senescence would be useful. Cellular senescence is a permanent state of cell cycle arrest in response to endogenous and exogenous stressors, such as telomere shortening and DNA damage due to radiation, chemical agents, reactive oxygen species (ROS), or other stressors (Di Micco et al. 2021; Huang et al. 2022). Senescent cells tend to accumulate with aging (Chaib et al. 2022), likely due to increased stressors with age and decreased clearance. Senescence burden can disrupt tissue functionality and limit the regenerative potential of adult stem cells (Moiseeva et al. 2023) and is theorized to drive poor health outcomes and chronic diseases with aging (L. Zhang et al. 2022). In line with the geroscience hypothesis, senolytic drugs have been developed to selectively target and kill senescent cells to reduce these age‐related risks. While these drugs have not been approved for clinical use, early trials in idiopathic pulmonary fibrosis and diabetic kidney disease suggest potential clearance of senescent cells and improved health outcomes in humans (Justice et al. 2019; Hickson et al. 2019). Clinical trials are currently underway for use of senolytics in treating and preventing diabetes, Alzheimer's, COVID‐19, osteoarthritis, and many more age‐related conditions (Chaib et al. 2022). Epigenetic clocks that capture cellular senescence could serve as early indicators of efficacy in clinical trials of senolytics and help determine if other prescribed drugs in clinical trials and population health studies may have senolytic properties.

However, current epigenetic clocks either show no change or inconsistent increases with senescence in vitro. Kabacik et al. (2022) found that the Horvath skin‐and‐blood (Horvath et al. 2018), Horvath pan‐tissue (Horvath 2013), Hannum (Hannum et al. 2013), and PhenoAge (Levine et al. 2018) clocks increased with replicative senescence but not radiation‐induced or RAS oncogene‐induced senescence. In Lowe et al. (2016), only replicative‐ and oncogene‐induced senescence are associated with increases in the Horvath pan‐tissue clock; neither radiation‐induced senescence nor oncogene‐induced senescence increased epigenetic age (Raj and Horvath 2020). Liu et al. (2020) similarly found that the PhenoAge clock increased with replicative and oncogenic senescence. However, the Lin clock (Lin and Wagner 2015) increased with replicative senescence only, and Hannum increased with oncogene‐induced senescence only but paradoxically showed a decrease with replicative senescence. All other clocks tested, including the Horvath pan‐tissue clock, were not associated with either type of senescence. Lu et al. (2018) found that immortalized cell lines increased in Horvath pan‐tissue clock epigenetic age linearly with proliferation, while non‐immortalized cells that reached senescence plateaued in epigenetic age. This suggests that the Horvath clock is capturing replication rather than replicative senescence per se. This may reconcile other reports where accelerated Horvath age was found, as earlier passage cells are compared to replicative senescent cells and thus could be explained by replication and not senescence. This is also consistent in Sturm et al. (2019), the authors found that in vitro human fibroblasts show increasing epigenetic age with passaging according to the Horvath pan‐tissue, Horvath skin‐and‐blood, PhenoAge, and Hannum clocks. However, only the skin‐and‐blood clock showed continued increases in epigenetic aging in replicative senescence; the rest of the clocks plateaued with senescence, further indicating that some clocks, including the Horvath pan‐tissue clock, may be capturing replication and not replicative senescence. In summary, current studies are inconsistent, no epigenetic clocks show reliable changes in all senescence types, and it is important to disentangle senescence‐related changes from other processes such as replication.

Even though some clocks may be associated with increased senescence burden, senolytics do not decelerate biological age according to any of the current epigenetic clocks in vitro (Boroni et al. 2020). This may reflect the differences between in vitro senescence and the in vivo settings in which the clocks were trained. However, two recent human clinical trials of the senolytics dasatinib and quercetin, with or without fisetin, given over 6 months also did not find any change in numerous epigenetic clocks, and in fact, several epigenetic clocks accelerated at the 3‐month time point (E. Lee et al. 2024).

Thus, per the existing literature, current epigenetic clocks are not consistently accelerated with cellular senescence and are not decelerated by senolytic drugs. Some have proposed that epigenetic aging and cellular senescence are largely independent processes (Raj and Horvath 2020). However, epigenetic clocks were trained based on age‐related methylation changes regardless of the underlying biology, and only a fraction of CpGs in current epigenetic clocks might be related to senescence. Here, we hypothesized that a specific subset of the aging DNA methylome is altered in cellular senescence; these methylation sites would be better suited for epigenetic clocks that detect cellular senescence and the effects of senolytic treatment. To test this hypothesis, we systematically analyzed CpG associations with cellular senescence, age, and mortality. This allowed us to focus on a smaller subset of the methylome to train multiple senescence‐enriched epigenetic clocks and test their response to senolytic treatments.

## 2 Results

### 2.1 A Limited Subset of CpGs Are Associated With Cellular Senescence

To identify CpGs associated with senescence, we performed a meta‐analysis of CpGs significantly altered in vitro (either hypomethylated or hypermethylated) by replicative, DNA damage, and oncogene‐induced senescence, utilizing four cell lines across two datasets (GSE227160 and GSE197723). We initially considered 396,333 CpGs found across all training and validation datasets (Methods). Information about cell lines and types of senescence in each dataset can be found in Table 1.

**TABLE 1:** In vitro senescence datasets.

In vitro senescence datasets								
	Dataset	Cell type	Array type	# Control	# Immortalized control	# Replicative	# Radiation	# Oncogene
Senescence training 1	GSE197723 (Experiment 1)	Fibroblasts	EPIC	113	0	24	24	24
Senescence training 2	GSE227160	Diploid skin fibroblasts, bone marrow MSC, adipose derived MSC	EPIC	12	0	12	24	0
Senescence testing 3	GSE37066	MSC	450k	5	0	5	6	0
Senescence testing 4	GSE91069	Fibroblasts	450k	6	12	6	0	3
Senescence testing 5	GSE197723 (Experiment 2)	MSC	EPIC	29	0	0	24	0
Senescence testing 6	GSE58035 (Experiment 1)	Epithelial cells	450k	3	0	3	0	0
Senescence testing 7	GSE58035 (Experiment 2)	Epithelial cells	450k	3	0	3	0	0

In addition to identifying senescence‐associated CpGs, we removed CpGs associated with the passaging of immortalized cells, which replicate indefinitely without entering senescence. Previous literature has found that replicative senescence is associated with many CpG changes and some epigenetic clock changes, more so than other types of senescence (Kwiatkowska et al. 2023; Kabacik et al. 2022). CpG methylation changes captured by clocks could, therefore, not be due to replicative senescence per se, but rather replication itself. Indeed, methylation changes can be used to track cumulative population doublings or time in culture (Koch and Wagner 2013; Minteer et al. 2023; Endicott et al. 2022). Therefore, we used data from extensively passaged immortalized cells that do not reach senescence in GSE226079 to find CpGs that are specifically correlated with replication. CpGs that were positively or negatively correlated (|r| > 0.3) with the passaging of immortalized cells were removed from downstream analysis (Table S1). We annotated these CpGs by associated gene and gene region (Table S2). Functional analysis of these “immortalization CpGs” showed GO and KEGG term enrichment in membrane, developmental, and neuronal processes (Figure S1). Figure S2 shows a histogram of gene regions for these immortalization CpGs.

**FIGURE 1:**
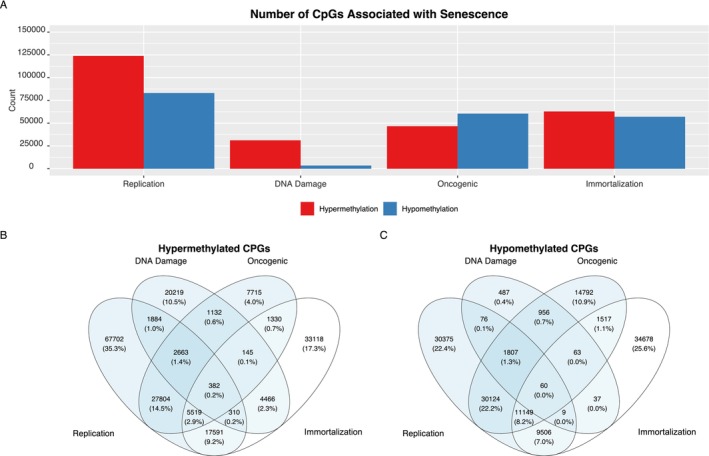
A small subset of CpGs are significantly hypermethylated and hypomethylated in senescence and immortalization. (A) Barplot illustrating the number of detected hypermethylated CpGs and hypomethylated CpGs in senescence (replicative, DNA damage, and oncogenic) as well as immortalization. (B, C) Venn diagrams illustrating overlaps between senescence‐related and immortalization‐related CpGs for hypermethylation (B) and hypomethylation (C) separately. Percentages are relative to the total number of CpGs identified in at least one hyper‐ or hypo‐methylation analysis, respectively, such that percentages for each Venn diagram adds up to 100%.

The number of hypermethylated and hypomethylated CpGs in each type of senescence is shown in Figure 1A, while the overlap of CpGs associated with senescence and immortalization is shown in Figure 1B,C. There were substantially more hypermethylated CpGs in DNA damage senescence (31,201) than hypomethylated (3495). Furthermore, there was a more modest hypermethylation bias for replicative senescence. In addition, in both hypermethylation and hypomethylation, the largest overlap in CpGs is in replicative and oncogene‐induced senescence (27,804 in hypermethylated and 30,124 in hypomethylated). Of the three types of senescence, immortalized CpGs unsurprisingly had the biggest overlap with replicative senescence (17,591 in hypermethylated and 9506 in hypomethylated).

These robust, shared methylation changes in senescence build on Kwiatkowska et al. (2023)'s findings that both the senescence induction mechanism—DNA damage versus replication—and cell line—human diploid skin fibroblasts (DSF), adipose‐derived mesenchymal stem cells (hAd‐MSC), or bone marrow‐derived mesenchymal stem cells (hBM‐MSC) can severely and independently bias the methylome towards hyper or hypo methylation. Consistent with Kwiatkowska et al. (2023)'s findings, both hypermethylation and hypomethylation, replicative senescent CpGs dominate in terms of amount, followed by oncogenic CpGs then DNA damage CpGs (Figure 1A). The cell type and senescence induction method shared methylation changes relate to Kwiatkowska et al. (2023)'s findings as their data is included in our analysis (GSE227160).

For downstream analysis, we decided to focus on CpGs that were significantly hyper‐ or hypo‐ methylated in at least 2 of the 3 types of senescence, while still keeping the same directionality in the 3rd type of senescence. This increased our confidence that we are looking at CpGs specifically related to a pan‐senescence phenotype, rather than an artifact specific to one senescence‐induction experiment. Overall, this resulted in a total of 37,815 CpGs—29,127 hypermethylated and 8688 hypomethylated for further analysis.

### 2.2 Few Senescence Related CpGs Are Associated With Age or Mortality

**FIGURE 2:**
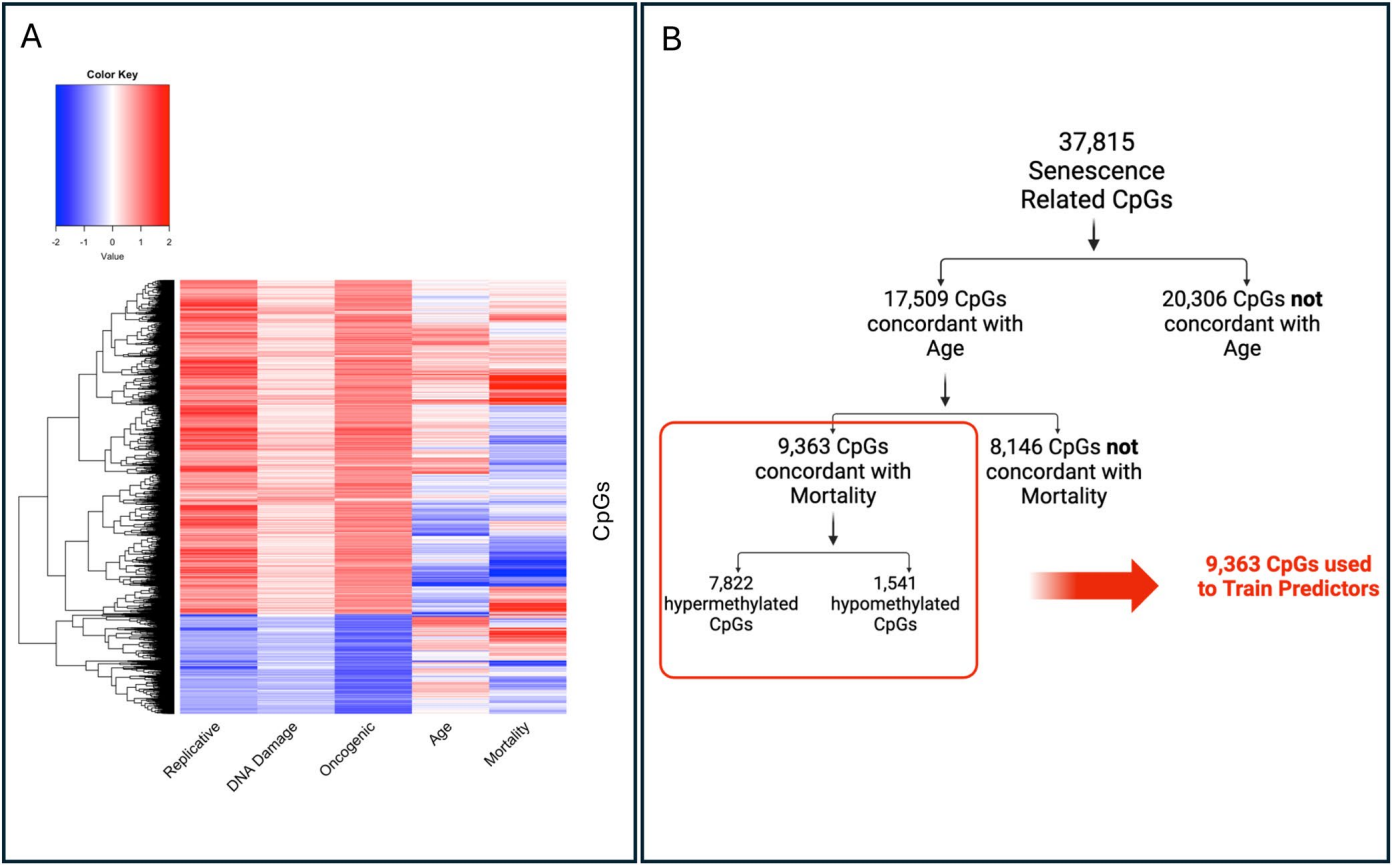
Many senescence‐related CpGs show null or opposite effects as age and mortality. (A) Clustered heatmap displaying the relationship between different CpGs (*y*‐axis) and different phenotypes of interest. Heatmap values for replicative, DNA damage, and oncogenic senescence are the standardized mean differences in *β*‐values for each CpG of senescent versus control samples. Heatmap values for age represent the age slope for the CpG, calculated as the SD change in CpG beta‐value for a 1‐SD change in chronological age. Heatmap values for mortality represent the standardized hazard ratio, the increased odds of mortality for a 1 SD change in CpG value. (B) Pipeline for selecting CpG for our models. Only the 9363 CpGs that were concordant with senescence, chronological age, and mortality were fed into our elastic predictors to train our models.

Our next goal was to understand how these senescence‐related CpGs change with aging in vivo. Figure 2A shows a heatmap of CpG associations with senescence, chronological age, and mortality risk. Statistics for all CpGs can be found in Table S1. For age, we calculated each CpG's change with age (age slope) in GSE40279, a large whole‐blood dataset. We found 17,509 CpGs that changed in the same direction with senescence as with age (hypermethylated with both, or hypomethylated with both). However, 20,306 CpGs showed opposite effects for senescence and age (hypermethylated in one while hypomethylated in the other). We calculated standardized hazard ratios in the Framingham Heart Study (FHS), a large whole blood dataset with mortality follow‐up information for up to 10 years after DNA methylation measurement, for the 17,509 CpGs that changed in the same direction with senescence and age. Of note, these hazard ratios were calculated in the training split of FHS that we utilize later for training mortality predictors. From these, 9636 CpGs showed the same direction of association for senescence as mortality risk (CpGs hypermethylated with senescence showed increased mortality risk with higher methylation levels, while CpGs hypomethylated with senescence showed increased mortality risk with lower methylation levels). However, 8146 CpGs showed opposite associations for senescence and mortality risk. In total, out of 37,815 senescence‐related CpGs, there were only 9363 CpGs that moved in the same direction with senescence, age, and mortality, which was surprising given that cellular senescence increases with age. A description of this CpG sub‐setting can be found in Figure 2B.

### 2.3 Senescence‐Associated CpGs Can Be Used to Train Epigenetic Clocks

Given that many CpGs show opposite effects for senescence as they do for aging and mortality, it is not surprising that existing epigenetic clocks trained to predict chronological age or mortality do not consistently capture cellular senescence. Thus, we hypothesized that focusing on the 9363 CpGs that show changes in consistent directions for senescence, age, and mortality risk could produce epigenetic clocks more relevant to senescence. We used the 9363 chosen CpGs to train three predictors: a binary senescence predictor (SenCultureAge), an age predictor (SenChronoAge), and a mortality predictor (SenMortalityAge) (Table S3). Our senescence predictor was trained on the same in vitro senescence datasets that we used to choose our CpGs (GSE227160 and GSE197720), where each sample was labeled as senescent or control. Batch correction was used to correct for technical variation between these two datasets (Figure S3). Our age predictor was trained on GSE40279 and our mortality predictor was trained on the training split of FHS (70/30 training/test split), both of which are whole blood datasets. SenChronoAge and SenMortalityAge are trained using similar outcomes and data as existing epigenetic clocks (e.g., Hannum, GrimAge) that have not shown consistent changes with senescence or senolytic treatment in previous (Kabacik et al. 2022; E. Lee et al. 2024). Tables S3–S5 display a list of CpGs included in each predictor, annotated with associated gene and gene region.

Functional analyses of the CpGs chosen for SenCultureAge, SenChronoAge, and SenMortalityAge suggest these do capture cellular senescence. Many of the CpGs in each model are linked to senescence‐related genes according to the CellAge database (Avelar et al. 2020) (Table S4). Figure S2 shows a histogram of gene regions for each model as well as our baseline and immortalization CpGs, showing similar distributions. GO and KEGG terms show enrichment for signaling and metabolic processes, and SenCultureAge is associated with Chromatin Accessibility Complex (CHRAC) (Figure S4). Trait enrichment analysis shows enrichment for aging, smoking, alcohol, cognition, pollution, and various diseases such as chronic obstructive pulmonary disease, Crohn's disease, myopia, cancer, and type 2 diabetes (Figures S4–, S6).

**FIGURE 3:**
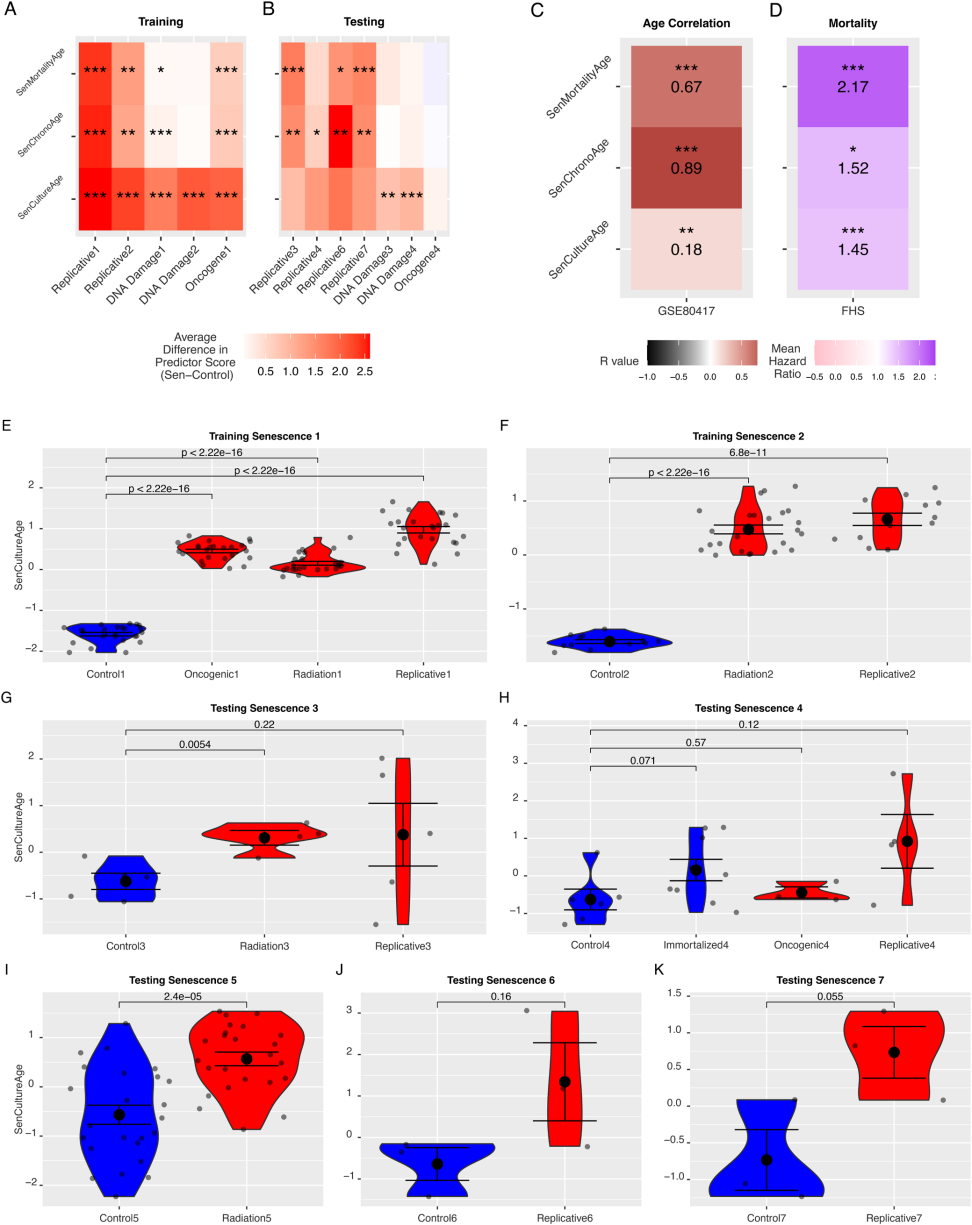
Senescence, age, and mortality predictors show significant changes in training and testing datasets. (A, B) Heatmap displaying differences between senescent and control samples in training datasets (A) and testing datasets (B) for 3 models (SenCultureAge, SenChronoAge, and SenMortalityAge). *x*‐axis refers to specific groups of senescence types and their corresponding control, separated by dataset and cell types to avoid differences due to batch effects. Each square represents the difference between mean predictor score in senescent samples and the mean predictor score in the corresponding control samples. Asterisks represent statistical significance, calculated with a two‐sample t‐test, and are denoted as follows: *** for *p* < 0.0001, ** for *p* < 0.001, and * for *p* < 0.05. (C) Heatmap displaying age correlation coefficient (r) in testing dataset for each predictor. (D) Heatmap displaying standardized hazard ratio in testing split of FHS dataset for each predictor. (E–K) Violin plots illustrating the difference in SenCultureAge predictor in senescence and controls in each individual training and testing dataset, with *p*‐values calculated using a two‐sample *t*‐test.

We tested each model's performance based on three criteria: (1) ability to differentiate between senescent and non‐senescent samples in both in vitro training (Figure 3A) and in vitro testing datasets (Figure 3B), (2) correlation with chronological age (Figure 3C), and (3) association with mortality risk (Figure 3D) in independent validation datasets not used for training. Information on all testing and training datasets can be found in Tables 1 and S5.

Our SenCultureAge score was accelerated in DNA damage senescence only (Figure 3A,B,E–K). In some datasets, the score was able to fully differentiate control and senescent cells with no overlap in scores. SenChronoAge was only accelerated in replicative senescence for both our training datasets and testing datasets. SenMortalityAge was accelerated in replicative and oncogenic senescence in our training datasets but was only accelerated in replicative senescence in some of our testing datasets. All three scores showed a positive correlation with age in our testing dataset, with SenChronoAge (*R* = 0.89) and SenMortalityAge (*R* = 0.67) showing stronger correlations than SenCultureAge (*R* = 0.18) (Figure 3C). All three scores were positively associated with mortality risk in the FHS testing split (Figure 3D), strongest for SenMortality Age followed by SenCultureAge. We termed these 3 scores “senescence‐enriched clocks.”

### 2.4 No Senescence‐Enriched Clock Was Altered by Senolytic Treatment

**FIGURE 4:**
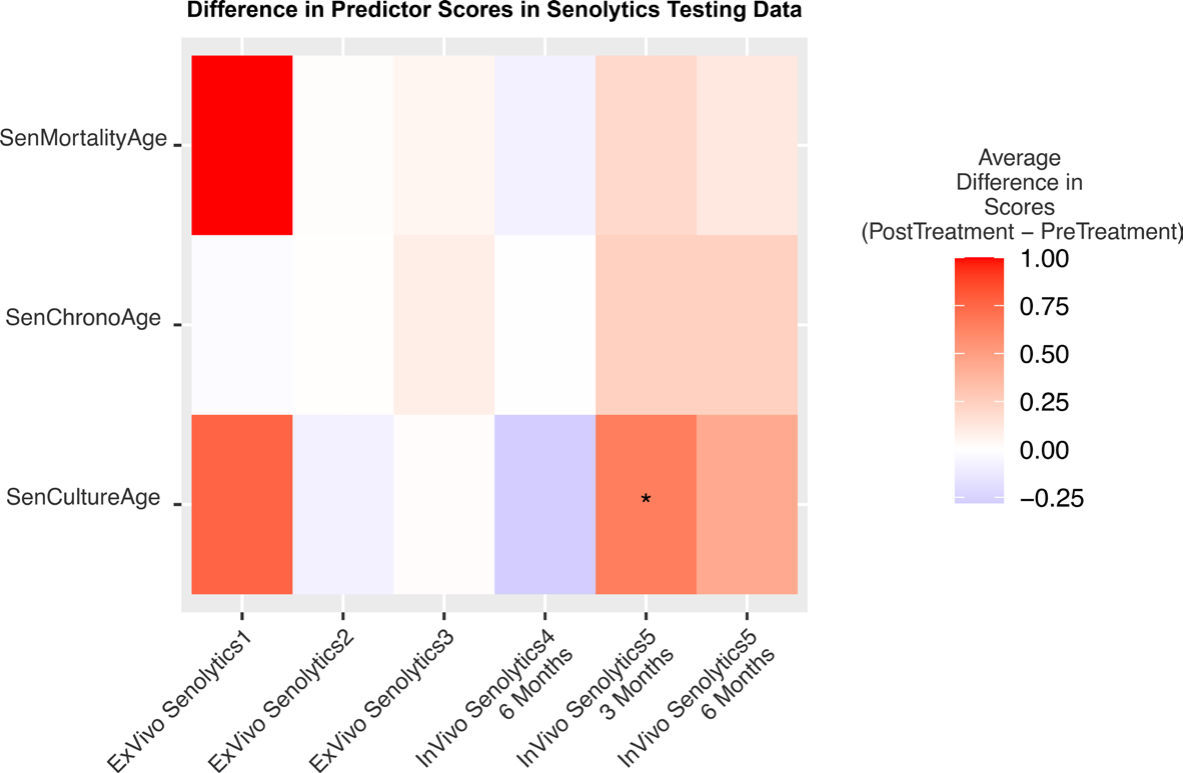
There are no consistent significant changes in senescence‐enriched clocks in pre and post treatment in vitro and in vivo senolytics. Heatmap illustrating the mean difference of all three predictors in post‐senolytic treatment samples and pre‐senolytic treatment samples. Asterisks represent statistical significance differences, calculated with a two‐sample *t*‐test, and are denoted as follows: *** for *p* < 0.0001, ** for *p* < 0.001, and * for *p* < 0.05.

Next, we examined how each senescence‐enriched clock responded to senolytic treatment in both in vitro and in vivo senolytics datasets (Figure 4). A summary of these datasets and senolytic treatments can be found in Table 2. Surprisingly, no senescence‐enriched clock showed a significant reduction for any in vitro or in vivo dataset. In fact, the scores trended towards accelerated values with senolytic treatment. The statistically significant change was SenCultureAge, which accelerated after 3 months of treatment with dasatinib and quercetin (DQ). Of note, multiple epigenetic clocks were previously shown to accelerate after 3 months of DQ treatment in this dataset (E. Lee et al. 2024).

**TABLE 2:** Ex vivo senolytics testing datasets and in vivo senolytics testing datasets.

Ex vivo senolytics testing datasets						
	Dataset	Cell/Tissue type	Type of senolytics	Array type	# of control	# of treated samples
Exvivosenolytics testing 1	GSE230026	Human fibroblasts	Pep14	EPIC	5	4
ExVivoSenolytics testing 2	GSE151617 (Experiment 1)	Human progeria fibroblasts	ABT 1.5um	EPIC	3	3
ExVivoSenolytics testing 3	GSE151617 (Experiment 2)	Human progeria fibroblasts	ABT 5um	EPIC	3	3
ExVivo mouse senolytics	GSE290909	Skeletal muscle tissue	BI01	Horvath 320k mammalian	33 (old, young, injured and uninjured samples ***not treated*** with senolytics)	18 (old, injured, and uninjured samples ***treated*** with senolytics)

In vivo senolytics testing datasets							
	Dataset	Tissue	Type of senolytics	Array type	# of samples	Age range	Time periods sampled
InVivoSenolytics testing 4	Tru diagnostic 1	Whole blood	Dasatinib 50 mg Quercetin 500 mg Fisetin 500 mg (DQF)	EPIC	19	42.95–86.55	6 months
InVivoSenolytics testing 5	Tru diagnostic 2	Whole blood	Dasatinib 50 mg Quercetin 500 mg (DQ)	EPIC	19	44.54–88.02	3 months, 6 months

### 2.5 Senescence‐Associated CpGs Are Inconsistently Modified With Senolytics

**FIGURE 5:**
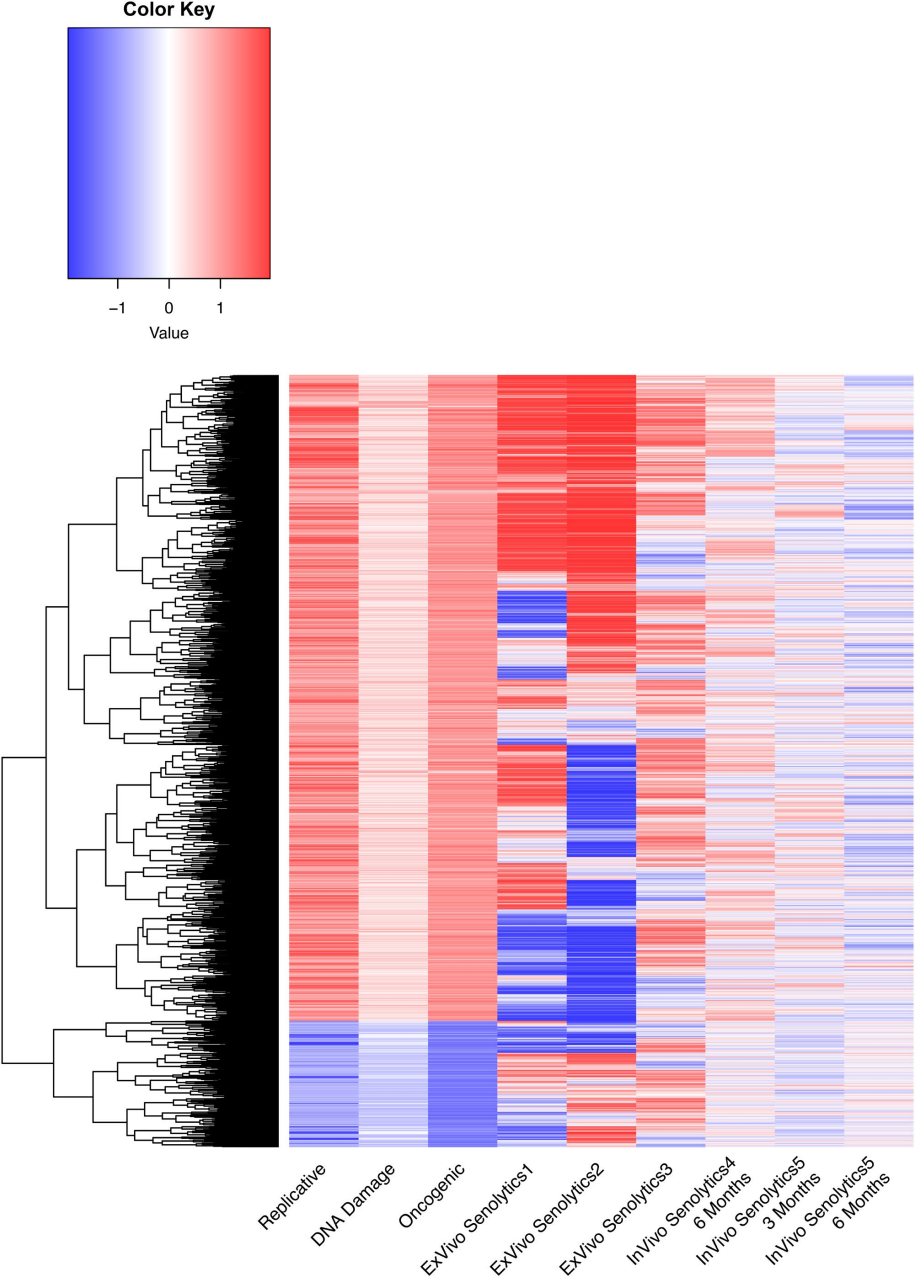
Changes in senescence related CpGs are not reversed by senolytics. Heatmap displaying effect sizes (scaled mean differences) for each senescence related CpG in both senescence (replication, oncogene, DNA damage) and in vitro and in vivo senolytics.

To better understand why our senescence‐enriched clocks did not decelerate with senolytics, we looked at the effect sizes for each of the 9363 CpGs, comparing the effects of each type of senescence and senolytic (Figure 5). Statistics for all CpGs can be found in Table S1. In all senolytic datasets, many CpGs changed in the same direction with senescence and senolytics, explaining some previously observed paradoxical accelerations in epigenetic clocks (Figure 4; (E. Lee et al. 2024)). Although there were some CpGs that changed in the opposite direction for senescence and some senolytic treatments, these were not consistent between datasets. Therefore, though we considered training a predictor based on CpGs changing in the opposite direction for senescence and senolytic treatment, this turned out to be infeasible.

### 2.6 Human‐Mouse Senescence‐Enriched Clocks Are Not Altered by Senolytic Treatment

In Chambers et al. (2025), the authors assess DNA methylation age with mouse epigenetic clocks (Mozhui et al. 2022), 35 days after healing from a BaCl2 injection in both young and old mice. During recovery, some samples were treated with senolytics (BI01). To test whether senolytic treatment reverses DNA methylation senescence signatures, we trained novel mouse‐human senescence‐enriched clocks, which is challenging as only 5081 CpGs overlap between our human data and the Mammalian 320 k array. To maximize the number of CpGs available for training, we utilized less stringent criteria, focusing on CpGs that were significantly hyper‐ or hypo‐ methylated in at least 1 of the 3 types of senescence, while keeping the same directionality with the other senescence inducers. This yielded 1081 CpGs (Figure S7). We used the same datasets and procedures to train human‐mouse models. These models were then applied to the Chambers dataset to test if any of our predictors change in: (1) uninjured versus injured young and old mice (2) old versus young control and injured mice (3) no senolytic versus senolytic treatment old control and old injured mice. We did not find any significant change in any of our models for any of our comparisons (Figures S8–, S10), potentially due to low overlap in CpGs.

### 2.7 Sensitivity Analysis: Training Predictors for Different Types of Senescence

In this study, we selected CpGs with significant changes across at least two types of senescence, aiming to identify universal markers of senescence and include CpGs more robustly associated with senescence. However, this approach may overlook epigenetic signatures associated with only one type of senescence inducer. To test this, we conducted a sensitivity analysis by training new predictors for each senescence inducer separately, using otherwise the same approach as prior (Figures S11–, S13). Overall, predictors utilizing replicative senescence CpGs or oncogenic senescence CpGs gave very similar results as our “universal” predictor, while predictors using DNA damage related CpGs only were significantly less responsive to all types of senescence (Figure S12C). Again, none of these predictors were responsive to senolytic treatment (Figures S11F–, S13F).

## 3 Discussion

Previous literature has not found epigenetic clocks to consistently accelerate with cellular senescence nor decelerate with senolytic treatment, posing the question of whether cellular senescence and epigenetic aging are, in fact, distinct phenomena. In this study, we decided to investigate this further by performing the first systematic comparison of CpG‐level changes with (1) cellular senescence in vitro, (2) age‐ and mortality‐associated CpGs in vivo, and (3) senolytics both in vitro and in vivo. We found only a small subset of CpGs are changed in the same direction as cellular senescence, age, and mortality risk (Figure 2), which may explain why prior epigenetic clocks show modest and inconsistent associations with senescence. By focusing on this smaller subset of the methylome, we could train novel epigenetic clocks associated with senescence, age, and mortality (Figure 3). Our results support the idea that we can focus on specific subsets of CpGs to capture specific aspects of aging, an approach taken by multiple recent studies (Ying et al. 2024; Ndhlovu et al. 2024). However, none of the clocks we developed responded to senolytics either in vivo or in vitro. Thus, even refined clocks may fail to capture senolytic effects, limiting their utility as clinical trial endpoints.

Prior work has aimed to identify “universal” signatures of senescence shared across cell types and senescence inducers (Wang et al. 2025), while others have attempted to find signatures specific to particular cell types or inducers (Sanborn et al. 2025; Cohn et al. 2023). Here, we focus on identifying universal markers, reasoning these would be most clinically relevant as in vivo age and disease related cellular senescence will likely involve multiple inducers and cell types. Furthermore, a universal signature would reduce multiple testing in future studies. Accordingly, we used datasets with a variety of cell types and multiple senescence inducers. Our analysis suggests cells share senescent epigenetic changes regardless of cell type, whereas senescent inducers generate distinct epigenetic signals with less overlap. This raised the possibility that different DNA methylation biomarkers should be trained for different senescence inducers. However, our sensitivity analyses found that replicative and oncogenic senescence predictors yielded similar results as the universal senescence predictors, while the radiation‐induced senescence predictor showed reduced associations. Thus, there does not currently appear to be any clear benefit to developing DNA methylation biomarkers for different cell types or senescence inducers, although this may change as new technologies are developed and new data is collected.

Surprisingly, most CpGs associated with in vitro senescence changed in the opposite direction with increased age and mortality in vivo (Figure 2). These results align with previous findings that epigenetic clocks—trained only on age and/or mortality‐related CpGs—did not show significant acceleration in all types of in vitro senescence. Possible explanations include: (1) Senescence may not always be associated with increased biological age or disease risk, as senescence drives positive biological functions such as wound healing and development (Huang et al. 2022; Khalid et al. 2022); (2) In vitro and in vivo DNAm senescent signatures are driven by different cells. In vitro, a large proportion of cells become senescent, whereas in vivo, a significantly smaller proportion of cells are senescent. It is possible that in vivo DNAm signatures originate from non‐senescent cells reacting to senescent cells in the environment, making the two signatures significantly different; (3) There may be a nonlinear relationship between aging and senescence, as we previously found that epigenetic clocks seem to initially accelerate in with cellular passaging, only to plateau and then decelerate (Higgins‐Chen et al. 2022). Together, these possibilities suggest that senescent cells may have a “younger” epigenetic signature in some cases.

Functional analysis of CpGs in senescence‐enriched clocks is consistent with capturing senescence biology. Many of the CpGs are located on the promoter region of senescence‐inhibiting genes, such as ALKBH3, ILK, RRP8, and SRC, and on the body region of senescence‐inducing genes, such as HIPK2, MAD1L1, and RELB (Tables S6–S8) (Avelar et al. 2020). SenChronoAge is associated with chromatin accessibility complex 1, and increased chromatin accessibility is a hallmark of cellular senescence (Lopes‐Paciencia and Ferbeyre 2025). Associated traits are known to be related to senescence, such as smoking, which upregulates the senescence‐related p53 and p16‐retinoblastoma protein pathways (Nyunoya et al. 2006). Other enriched traits with known relationships to senescence include myopia (Su et al. 2025), Crohn's disease (W. Zhang et al. 2025), aortic dissection (Nakao et al. 2024), and chronic pulmonary disease (Rivas et al. 2022).

Notably, senolytics failed to decrease the score of any of our senescence‐enriched clocks, either in vitro or in vivo, and in fact the changes trended towards acceleration (Figure 4). This agrees with prior findings that other clocks increase after 3 months of treatment with dasatinib and quercetin then return to baseline (E. Lee et al. 2024). These results are surprising, because the expectation is that senolytic treatment would clear senescent cells, and the remaining non‐senescent cells should have lower senescence scores and epigenetic ages. This raises important alternative hypotheses that should be explored in future studies: First, some senescent cells may be refractory to the particular senolytic drugs administered, as senolytic drugs differ in their mechanisms of targeting senescent cells (Rad and Grillari 2024). However, this is unlikely to be an adequate explanation, since none of the datasets analyzed showed reductions. Second, the senolytics may clear senescent cells, but they may simultaneously exert pro‐aging effects on the non‐senescent cells, as early trials have indicated cytotoxic effects with dose‐dependent usage (Raffaele and Vinciguerra 2022). These effects on senescent and non‐senescent cells may cancel each other out in terms of total senescence scores. Third, after the senolytics clear senescent cells then the remaining non‐senescent cells may increase their proliferation rate, which in turn increases the senescence scores which again would cancel out the effect of senescent cell clearance. Fourth, it may be that the DNAm senescence signature does not come from senescent cells at all, but rather from the non‐senescent cells reacting to the presence of senescent cells. In this case, the non‐senescent cells may not revert their DNAm to a low‐senescence state during the time course of the experiment. To address these different possibilities, future studies should focus on the effects of senolytics on the DNAm of non‐senescent cells.

Prior work by Chambers et al. supports the idea that DNA methylation may reflect the response of non‐senescent cells to nearby senescent cells. (Chambers et al. 2025). They found that in older mice, muscle injury followed by regeneration dramatically lowered muscle DNA methylation age (DNAmAge; up to 68%) over the course of 35 days, compared to uninjured older mice. Administration of a novel senolytic BI01 starting at day 13 after injury caused a modest further reduction in DNAmAge in injured mice, but not uninjured mice, and also results in better functional recovery in injured mice. The fact that both the increase of senescent cells by injury and subsequent clearance of senescent cells caused reductions in DNAmAge suggests this measure is not capturing the senescent cells themselves. Instead, the specific methylation changes induced by senolytic treatment were in collagen, Hox, and Wnt genes that suggest satellite cell proliferation which promotes regeneration. Their observation of reduced DNAmAge with senolytics could conceivably be due to reduced toxicity of the novel BI01 senolytic. Of note, they utilized a general DNA methylation aging biomarker, rather than a senescence‐enriched clock. We attempted to utilize our algorithms to better isolate senescence in this dataset and observed no changes with either injury or senolytic treatment. While one could posit this is evidence that senescence per se is not the mechanism by which epigenetic age is modulated in this experiment, we believe the interpretability of this analysis is limited due to the low overlap in methylation sites between human and mammalian arrays, which is the most likely explanation for no observed effect.

There are several limitations to our analysis. The in vivo senolytic datasets covered a relatively short 6‐month period, so delayed effects could have been missed. However, 6 months is a typical time scale for a clinical trial. One of the main reasons that the aging field is pursuing biomarkers is that they may provide early detection of treatment efficacy, long before age‐related loss of function or disease would manifest (Moqri et al. 2023). If epigenetic clocks take much longer than 6 months to detect senolytic effects, their practical utility diminishes. Another limitation to our findings is that the datasets used both to choose senescence‐related CpGs and train our SenCultureAge predictors were in vitro, and our results could be affected by in vitro artifacts. In vivo senescence datasets could provide more translatable signals consistent with senescence, age, and mortality. Previous research suggests that current epigenetic clocks, including Horvath, PhenoAge, GrimAge, and DunedinPoAm38, are very weakly related to senescence in vivo through correlation with the senescence marker p16 (Sedrak et al. 2023). Once clinical trial data combining in vivo senescence and DNA methylation data is made available, future studies should repeat our strategy of subsetting CpGs to enrich for senescence‐related signals for a more direct analysis.

We were also constrained by dataset heterogeneity: senescence induction protocols and senolytic treatments varied across studies. Ideally, the same samples would undergo both senescence induction and subsequent senolytic treatment, allowing direct within‐sample comparison. Nevertheless, our finding that universal senescence‐specific methylation signatures are not reversed by senolytics stands. This suggests a generalizable senescence biomarker that responds to senolytics, shared across multiple cell types and multiple senescence inducers, may be difficult to achieve, though DNA methylation biomarkers responsive to senolytic treatment may still be possible in very specific contexts.

Methylation data suffers from interpretability constraints given both cis‐ and trans‐ regulatory impacts on gene expression. Future work including paired gene expression data could provide a better understanding as to the functional impacts of these senescent DNA methylation changes (ex. whether promoter region methylation downregulates gene expression). These paired datasets could also explore the relationship between samples with “senescent” clock acceleration and expression of genes related to the senescence phenotype, such as Senescence‐Associated Secretory Phenotype (SASP).

### 3.1 Reverse Epigenetic Signatures

In summary, CpGs shared between senescence, aging, and mortality capture overlapping biology, but the relationships are complex. Clocks derived from overlapping CpGs do not reverse with senolytics, challenging the assumption that aging biomarkers must mirror rejuvenation. Further studies are needed to evaluate whether the difficulty in capturing intervention effects is true across different anti‐aging treatments or hallmarks of aging, or whether senescence is a specific case where the anti‐aging treatments do not reverse epigenetic signatures.

## 4 Methods

### 4.1 Datasets

The getGEO function from “GEOquery” R library version 2.68.0 (Davis and Meltzer 2007) was used to download pre‐processed beta values from all GEO datasets. All datasets are further detailed in Tables 1, 2, and S7.

### 4.2 In Vitro Senescence Datasets

#### 4.2.1 GSE197723

This dataset was previously described in Kabacik et al. (2022). Briefly, primary human dermal fibroblasts were isolated and divided into four groups: controls, replicative senescence, DNA damage senescence, and oncogenic senescence. To induce replicative senescence, cells were cultured for up to six months and were terminated when cells ceased proliferating after 14 days in culture with repeated media change. For X‐irradiation, cells were exposed to X‐rays with 250 kV, 13 mA and 60 cm from the source, giving a dose rate of 0.5 Gy min^−1^. Retroviruses carrying activated the RAS gene were transduced to induce oncogenic senescence.

#### 4.2.2 GSE227160

As described in Kwiatkowska et al. (2023), replicative senescence was induced in DSF, hBM‐MSC and hAd‐MSC with serial subculturing up to exhaustion of their proliferative potential. This corresponded to 65, 35, and 45 population doublings for DSF, hBM‐MSC, and hAd‐MSC respectively. DNA damage‐induced senescence was induced by exposing cells to either γ‐irradiation in a 60Co gamma source at a rate of 2.5 Gy/min or exposed to two non‐cytotoxic doses of doxorubicin (0.1 μΜ/dose) and proliferated until exhaustion. Senescence was confirmed by the inability of the cells to incorporate bromodeoxyuridine (BrdU) into their nuclei.

#### 4.2.3 GSE37066

As previously detailed in Koch et al. (2011), MSCs were isolated from the tibia plateau of patients undergoing orthopedic surgery. For DNA damage‐induced senescence, MSC were treated with a blood product irradiator with 15 Gy, and methylation profiles were analyzed seven days after treatment. Replicative senescence was induced by culturing cells until they entered growth arrest, after approximately 80 ± 25 days and 35.1 ± 5.7 cumulative population doublings. Senescence was confirmed by the acquisition of large and flat morphology and expressed senescence‐associated beta‐galactosidase (SA‐β‐gal).

#### 4.2.4 GSE91069

This dataset was previously described by Xie et al. (2018). Human foreskin fibroblasts were obtained, and oncogenic senescence was induced by infecting cells with retrovirus encoding HrasV12. Replicative senescence was induced by continuous culturing cells (approximately 28 population doublings) until they ceased to proliferate. Senescence was confirmed with β‐galactosidase staining assay.

#### 4.2.5 GSE58035

As detailed by Lowe et al. (2015), normal human mammary epithelial cells were obtained from a healthy 21‐year‐old female donor. Cell cultures were serially passaged until reaching replicative senescence, which was defined as the cells undergoing further expansion upon at least two serial passages. Senescent cells were checked for elevated expression of p16INK4A (p16) and SA‐β‐gal.

### 4.3 In Vitro Immortalized Datasets

#### 4.3.1 GSE226079

This dataset was previously described by Minteer et al. (2023).

Human telomerase reverse transcriptase (hTERT)‐immortalized fetal astrocytes were serially passaged until p27, and DNAm was assessed longitudinally at each passage using the Infinium HumanMethylation850 BeadChip. These cells showed no signs of growth arrest, genomic instability, or telomere erosion after 73+ cumulative population doublings.

### 4.4 In Vivo Age Datasets

#### 4.4.1 GSE40279

The experimental procedures for this study have been previously described in Hannum et al. (2013). Blood was drawn from 656 participants (426 Caucasian and 230 Hispanic) ages 19–101. Illumina Infinium HumanMethylation450 BeadChip was employed to assay DNA methylation. The study was approved by the institutional review boards of the University of California, San Diego, the University of Southern California, and West China Hospital. All participants signed informed consent.

#### 4.4.2 GSE80417

As previously detailed in Hannon et al. (2016), whole blood data was derived from the University College case–control sample, which includes 353 schizophrenia cases and 322 controls ages 18–87. Data was used from both the control and schizophrenia subjects.

### 4.5 In Vivo Mortality Datasets

#### 4.5.1 Framingham Heart Study (FHS)

The Offspring and Third Generation cohorts of FHS have previously been described (Markov et al. 2024; Higgins‐Chen et al. 2022; Kannel et al. 1979; Splansky et al. 2007). The FHS offspring cohort includes 2748 participants attending the eighth exam cycle (2005–2008), and the Third‐Generation cohort includes 1457 participants attending the second exam cycle (2005–2008). DNA methylation was assayed with the Infinium HumanMethylation450 BeadChip. For this study, all causes of death were reviewed by an endpoint panel of three investigators. All participants provided written informed consent at the time of each examination visit, and the study protocol was approved by the IRB at Boston University Medical Center.

### 4.6 In Vitro Senolytics Datasets

#### 4.6.1 GSE230026

This dataset was previously described in Zonari et al. (2023). DNA samples were obtained from human skin biopsy samples (Female, Caucasian, 79 years) and applied to the human Illumina Infinium EPIC 850 K chip. To induce accelerated aging, the cells were treated for 24 h with 20 μM etoposide and were removed after two days. Cells were then treated for 48 h with 12.5 μM of Pep 14. Pep 14 works by modulating genes that prevent senescence progression by arresting the cell cycle and enhancing DNA repair, reducing the number of cells that progress to senescence (Zonari et al. 2023). Senescence levels were then determined by SA‐βGal staining and normalized to total cell number.

#### 4.6.2 GSE151617

This dataset was previously described by Boroni et al. (2020). Primary human dermal fibroblasts from a six‐year old Hutchinson‐Gilford Progeria HGPS donor were obtained from The Progeria Research Foundation Cell and Tissue Bank. For senolytic treatment, ABT‐263 at a concentration of 1.25 or 5 μm was added to cell culture media and maintained for three days. ABT‐263 is an inhibitor of the anti‐apoptotic proteins BCL‐2 and BCL‐xL, which induces apoptosis (Chang et al. 2016). Human Illumina Infinium EPIC 850K chip was employed to assay DNA methylation.

### 4.7 In Vitro Senolytics Datasets: Mouse Model

#### 4.7.1 GSE290909

This dataset was previously described by Chambers et al. (2025). Briefly, male mice were split into two groups‐ injured mice (via 10 μL of 1.2% BaCl_2_ injection) versus controls (phosphate buffered saline injection). Both the control and injured mice had 3 groups: young mice (5–6 month) without senolytic treatment, old mice (24–25 month) without senolytic treatment, and old mice with novel senolytic treatment BI01, which works by inhibiting MDM2‐p53 binding, activating p53, and triggering apoptosis in senescent cells (Nolt et al. 2024). Administration of this senolytic occurred on Days 13, 14, 15, 20, 21, and 22 after injury. On Day 35 after injury, the tibilalis anterior was removed and methylation assayed using the Horvath 320K Mammalian Methylation array.

### 4.8 In Vivo Senolytics Datasets

#### 4.8.1 TruDiagnostic DQ and DQF

The details of this clinical trial are further detailed in Lee et al. (Valentim et al. 2024). For the DQ treatment cohort, 19 study participants were selected from November 2020 to December 2020 at the Institute for Hormonal Balance, Orlando. Informed consent was obtained from study participants, and the FDA‐registered IRB (Institute for Regenerative and Cellular Medicine) approved this study. Subjects were treated with 500 mg Quercetin and 50 mg Dasatinib oral capsules on Monday, Tuesday, and Wednesday (3 days in a row) per month for 6 months. While Dasatinib is a tyrosine kinase inhibitor approved by the FDA to treat myeloid leukemia, Quercetin is a flavonoid compound that induces apoptosis in senescent endothelial cells; the two together have been shown to clear senescent cells by inducing apoptosis (Kirkland and Tchkonia 2020). Whole blood was collected at baseline, at 3 months, and at 6 months. For the DQF trial, ten participants from the DQ study and nine patients recruited from June 2022 to July 2022 at the Institute for Hormonal Balance, Orlando, were included in the study. Subjects were treated with the same dosage and timeline as in the DQ study, but this trial also included 500 mg of Fisetin oral capsules on Monday, Tuesday, and Wednesday (3 days in a row) per month. Eight participants got a strawberry based Fisetin and 11 got a non‐strawberry based Fisetin. Infinium HumanMethylationEPIC BeadChip was used to assay DNA methylation.

### 4.9 Statistical Analysis

Data processing and analysis were performed using R version 4.3.1. For all *t*‐tests performed, normal distributions were assumed but not tested.

### 4.10 Selecting Senescence‐Related CpGs

We analyzed each type of senescence for each cell line separately, calculating effect sizes (scaled mean differences) and *p*‐values comparing senescent cells to control cells for each cell line. Based on the sign of the effect sizes, we separated CpGs into those hyper‐ and hypomethylated in each type of senescence. For replication‐ and DNA damage‐induced senescence, where we had data from multiple cell lines, we performed a meta‐analysis to summarize associations as a single effect size and *p*‐value. Multiple testing corrections were performed using the Bonferroni method.

For this analysis, we only considered the 396,333 CpGs present in all of our in vitro and in vivo testing and training datasets. To identify senescence‐associated CpGs (either significantly hypermethylated or hypomethylated), the glm function from “stats” R library version 4.3.1 was used to create a generalized linear model for each CpG in each senescence type, with the scaled methylation values for each CpG as the independent variable and senescent status (1—senescent, 0—control, NA—for all other types of senescent not being analyzed) as the dependent variable. Beta values for each CpG were scaled by centering and dividing by the standard deviation to account for differences in variance for each CpG. CpGs with mean differences (average change in the log odds of the beta value associated with a change to senescent state) > 0 and *p* < 0.05 were considered significantly hypermethylated in senescence, and CpGs with mean differences < 0 and *p* < 0.05 were considered significantly hypomethylated. When we had data from multiple cell lines as in replication and DNA damage‐induced senescence, we generated a single mean difference and *p*‐value by performing a fixed‐effects meta‐analysis weighted by inverse variance (C. H. Lee et al. 2016). For downstream analysis, we chose CpGs that were significantly hypomethylated or hypermethylated in at least two types of senescence, while maintaining the same directionality in the third type of senescence.

### 4.11 Removing Immortalized Related CpGs

To find CpGs associated with immortalized passaging, bi‐weight mid‐correlation was calculated between beta values and the passage number in GSE226079 using the function bicorAndPvalue from “WGCNA” R library version 1.72–5. CpGs with correlation |r| > 0.3 with a Benjamini‐Hochberg corrected *p*‐value < 0.05 were considered either significantly hypermethylated or hypomethylated with replication under immortalized conditions and were removed from downstream analysis.

### 4.12 Age Slope and Hazard Risk Ratio

To calculate CpG associations with age, glm was used to create a generalized linear model measuring the change of scaled beta values for each CpG with chronological age. Association with mortality was assessed via Cox proportional hazard regression models using R package survival (version 3.5.7). Models were standardized, meaning that hazard ratios represent the effect of a 1 standard deviation (SD) change in beta value, and they were adjusted for chronological age.

### 4.13 Functional Analysis of CpGs

CpG sites included in SenCultureAge, SenChronoAge, SenMortalityAge, and immortalization related CpGs were annotated using the R packages IlluminaHumanMethylationEPICanno.ilm10b4.hg19 (version 0.6.0) and Minfi (version 1.54.1); GO and KEGG analysis on these CpGs were performed using the R package missMethyl (version 1.42.0). Trait enrichment analysis on these CpGs was performed using the EWAS Open Platform (NGDC), an online toolkit for epigenome wide association studies (Xiong et al. 2022). The EWAS atlas can be accessed at the following link: https://ngdc.cncb.ac.cn/ewas. Information about senescence related genes was curated from the CellAge database (Avelar et al. 2020), which can be accessed at the following link: https://genomics.senescence.info/cells/.

### 4.14 Building Predictors

Further information about each model can be found in Table S2.

#### 4.14.1 SenCultureAge

SenCultureAge was trained with the pooled data from GSE197723 and GSE227160 (details in Table 1). Before pooling the data, we used principal component analysis (PCA) to compare signals from the datasets and determine whether batch correction was needed before applying a supervised machine learning approach. After seeing that fibroblast samples from GSE197723 clustered separately from the fibroblast samples in GSE227160 (Figure S1), the function ComBat from R package “SVA” version 3.48.0 was used to perform batch correction on our pooled dataset. The principal components for this corrected data are plotted (Figure S1), and we subsequently used the corrected data to train a senescence predictor.

To train SenCultureAge as a senescence predictor, we utilized an elastic net regression with the glmnet function in the R package “GLMNet” R library version 4.1–8 (Friedman et al. 2010) fitted to a binomial family. The elastic net ⍺ parameter was set to 0.5. The lambda value, or the regularization parameter, was determined using 10‐fold cross‐validation on the training dataset. All missing values in our training and testing datasets were imputed using mean imputation.

#### 4.14.2 SenChronoAge

SenChronoAge was trained as a chronological age predictor with in vivo whole blood data from GSE40279 (details in Table 1). Elastic net regression was implemented with glmnet, using 10‐fold cross‐validation to determine our lambda parameter. Mean imputation was used to fill in any missing CpG values.

#### 4.14.3 SenMortalityAge

SenMortalityAge was trained as a mortality predictor with in vivo whole blood data from the training split of FHS (details in Table 1). Elastic net Cox regression was used to train a model of time‐to‐death. The lambda value was determined using 10‐fold cross‐validation on the training dataset. Mean imputation was used to estimate missing values.

## Author Contributions

A.T.H.‐C. conceived the project and study design. A.T.H.‐C., R.S., Y.M., J.G., and J.K. developed the analysis pipeline and identified relevant datasets. J.K. trained and validated the models. A.T.H.‐C. and J.K. wrote the manuscript. All authors edited the manuscript for submission.

## Funding

This work was supported by grants to AHC by the National Institute on Aging (NIA: 1R01AG065403). This work was also supported by NIH 5T15LM007056‐38.

## Conflicts of Interest

A.T.H.‐C. received consulting fees from TruDiagnostic and FOXO Biosciences for work unrelated to the current manuscript. R.S. is a Scientific Advisor for TruDiagnostic and has received consulting fees from the company. R.S. is a Director at Healthy Longevity Clinic and has received consulting fees for this position. R.S. has received consulting fees from the LongevityTech.fund, and Cambrian BioPharma unrelated to this publication. R.S. and A.T.H.‐C. are named as inventors on a patent for epigenetic clocks unrelated to the current manuscript.

## Supporting information


**Figure S1:** GO and KEGG enrichment analysis on all “immortalization” CpGs.

**Figure S2:** Histogram of gene location for 3 models (SenCultureAge, SenChronoAge, SenMortalityAge), immortalization CpGs, and 396,333 “baseline” CpGs.

**Figure S3:** Batch correction on GSE197723 and GSE227160 training datasets to remove technical variation before training our models.

**Figure S4:** Trait, GO, and KEGG enrichment analysis for 141 CpGs in SenCultureAge.

**Figure S5:** Trait, GO, and KEGG enrichment analysis for 188 CpGs in SenChronoAge.

**Figure S6:** Trait, GO, and KEGG enrichment analysis for 89 CpGs in SenMortalityAge.

**Figure S7:** (A) Pipeline for selecting CpGs for our mouse senescence model. Figure 7B and C. Venn diagram illustrating overlaps between senescence‐related and immortalization‐related CpGs for hypermethylation (B) and hypomethylation (C) in mouse‐senescence models.

**Figure S8:** SenCultureAgeMouse predictor in Chambers et al. (2025) dataset, displaying change in predictor in control versus injury mice in old and young mice, old versus young mice in control and injured mice, and no senolytic treatment versus senolytic treatment in old control and old injured mice.

**Figure S9:** SenChronoAgeMouse predictor in Chambers et al. (2025) dataset, displaying change in predictor in control versus injury mice in old and young mice, old versus young mice in control and injured mice, and no senolytic treatment versus senolytic treatment in old control and old injured mice.

**Figure S10:** SenMortalityAgeMouse predictor in Chambers et al. (2025) dataset, displaying change in predictor in control versus injury mice in old and young mice, old versus young mice in control and injured mice, and no senolytic treatment versus senolytic treatment in old control and old injured mice.

**Figure S11:** Sensitivity Analysis for Replicative senescence. (A) Pipeline for selecting replication senescence CpGs for sensitivity analysis. Figure S11B,C Heatmap displaying differences between senescent and control samples in training datasets (B) and testing datasets (C) for 3 models (SenCultureAge, SenChronoAge, and SenMortalityAge). Figure S11D. Heatmap displaying age correlation coefficient (r) in testing dataset for each predictor. Figure S11E. Heatmap displaying standardized hazard ratio in testing split of FHS dataset for each predictor. Figure S11F Heatmap illustrating the mean difference of all three predictors in post‐senolytic treatment samples and pre‐senolytic treatment samples.

**Figure S12:** Sensitivity analysis for DNA damage senescence. Pipeline for selecting CpGs (A), predictor results in training (B) and testing (C) in vitro senescence, age correlation (D), hazard ratio (E), and senolytics validation (F).

**Figure S13:** Sensitivity analysis for Oncogenic senescence. Pipeline for selecting CpGs (A), predictor results in training (B) and testing (C) in vitro senescence, age correlation (D), hazard ratio (E), and senolytics validation (F).

**Data S1:** acel70430‐sup‐0015‐Tables.xlsm.

## Data Availability

The senescence training (GSE227160 and GSE197723), senescence testing (GSE91069, GSE37066, GSE58035, and GSE197723), in vitro and mouse senolytics (GSE230026, GSE151617 and GSE290909), age correlation training (GSE40279), and age correlation testing (GSE80417) are all available for download on NCBI Gene Expression Omnibus (GEO) (https://www.ncbi.nlm.nih.gov/geo/). Beta values and phenotypic data from FHS are available in dbGaP (accession no. phs000724.v7.p11). In vivo senolytics data was provided by TruDiagnostic and can be provided upon request. Code to calculate senescence‐enriched clocks is available at https://github.com/HigginsChenLab/methylCIPHER.
