# Supplementary figures and images for: DNA Methylation Signatures of Cellular Senescence Are Not Reversed by Senolytic Treatment
Source: Aging Cell. 2026 Feb 26;25(3):e70430. doi: 10.1111/acel.70430 (PMC12938503; doi:10.1111/acel.70430)

# Immortalization CpGs- GO and KEGG Terms

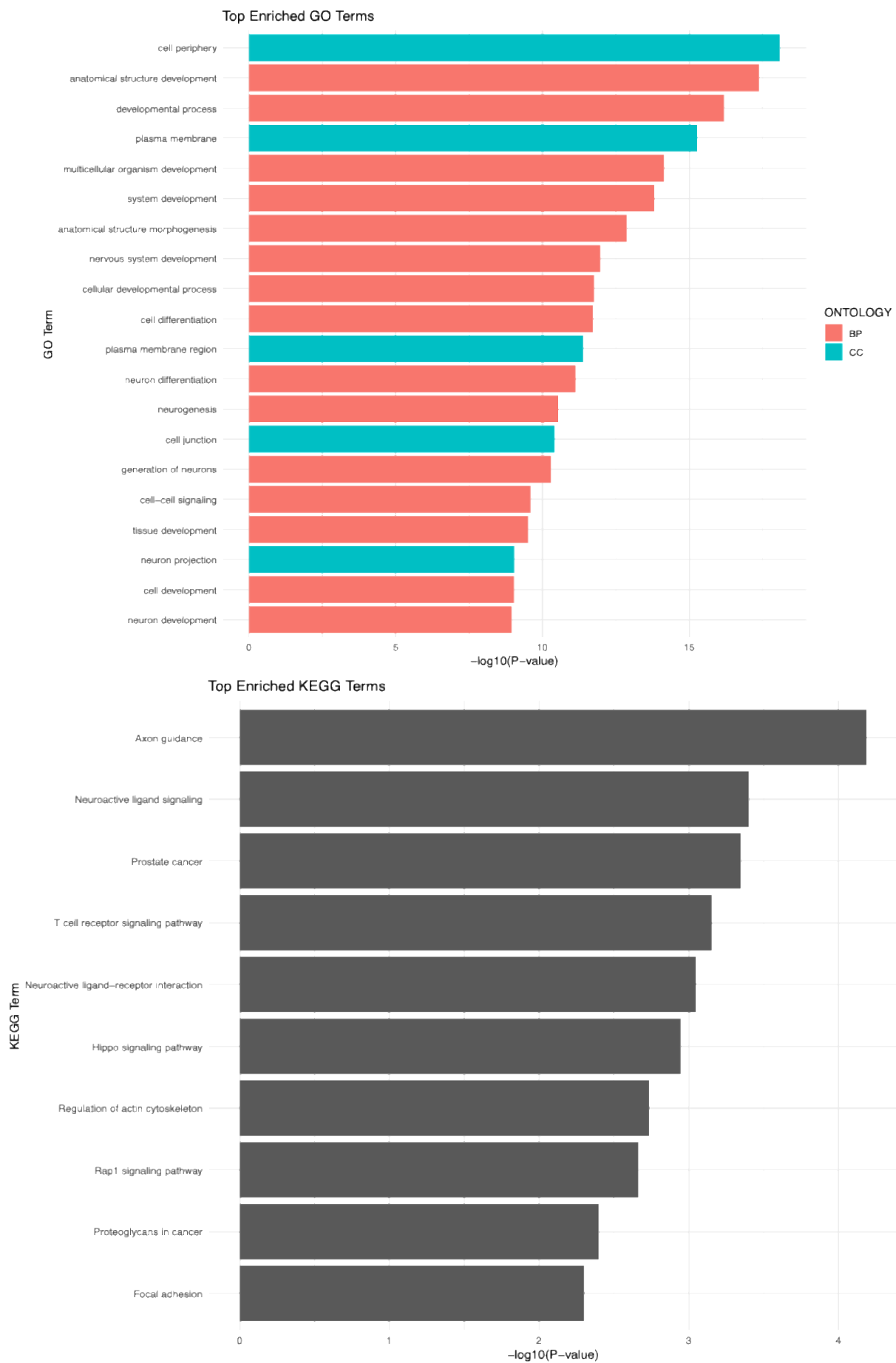

Supplement: Supplementary file 1 — Figure S1: GO and KEGG enrichment analysis on all “immortalization” CpGs. [file ACEL-25-e70430-s009.pdf]

CpG Location Distribution

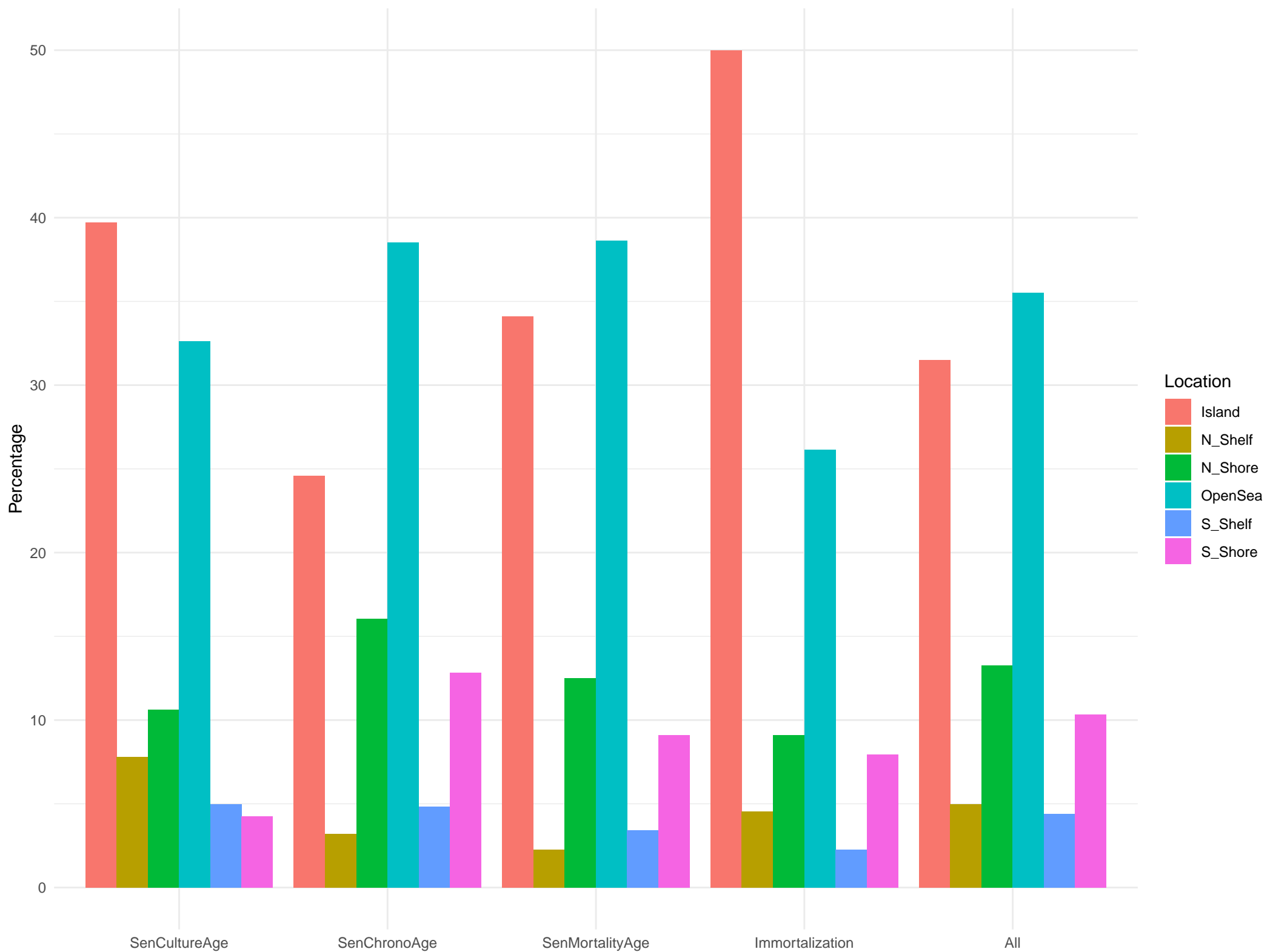

Supplement: Supplementary file 2 — Figure S2: Histogram of gene location for 3 models (SenCultureAge, SenChronoAge, SenMortalityAge), immortalization CpGs, and 396,333 “baseline” CpGs. [file ACEL-25-e70430-s006.pdf]

A

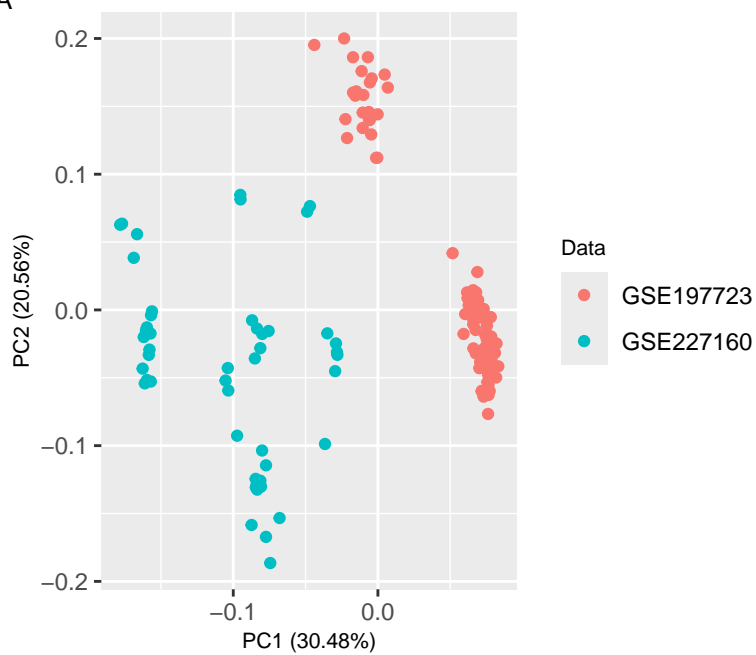

B

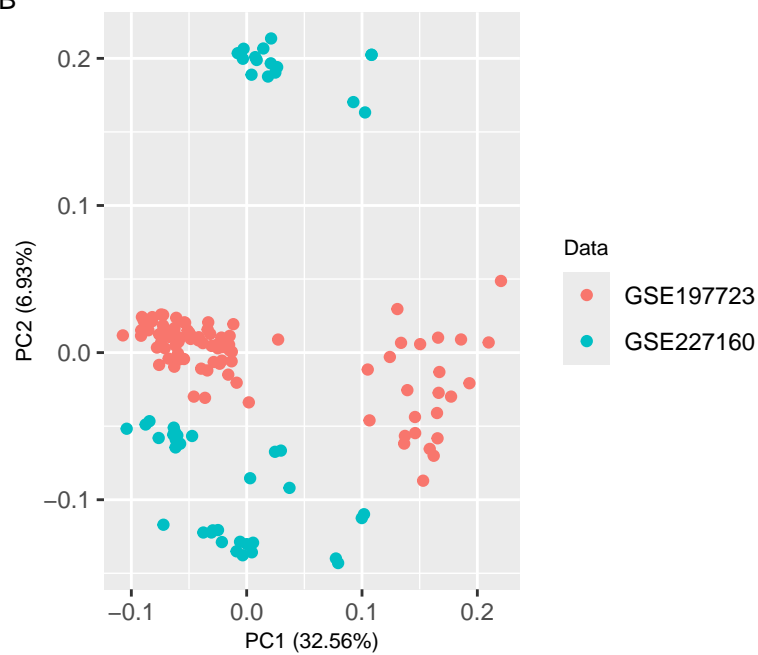

C

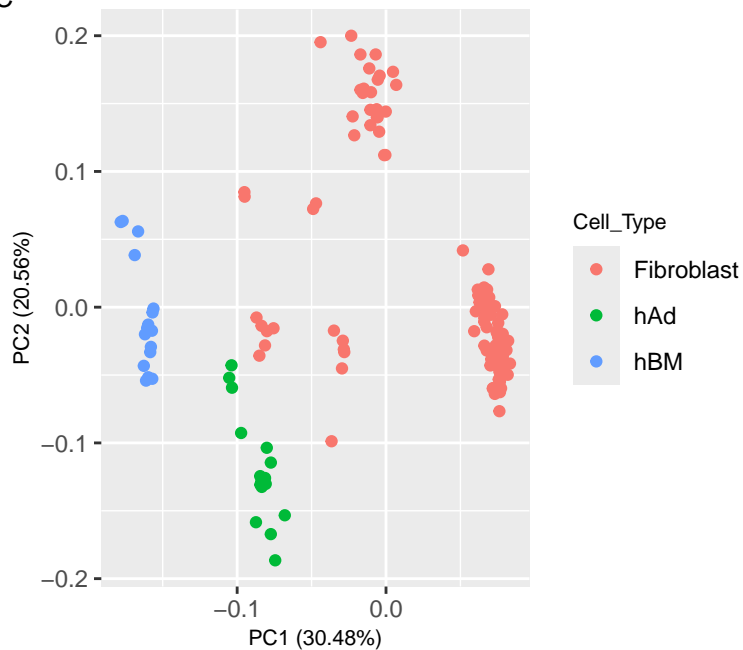

D

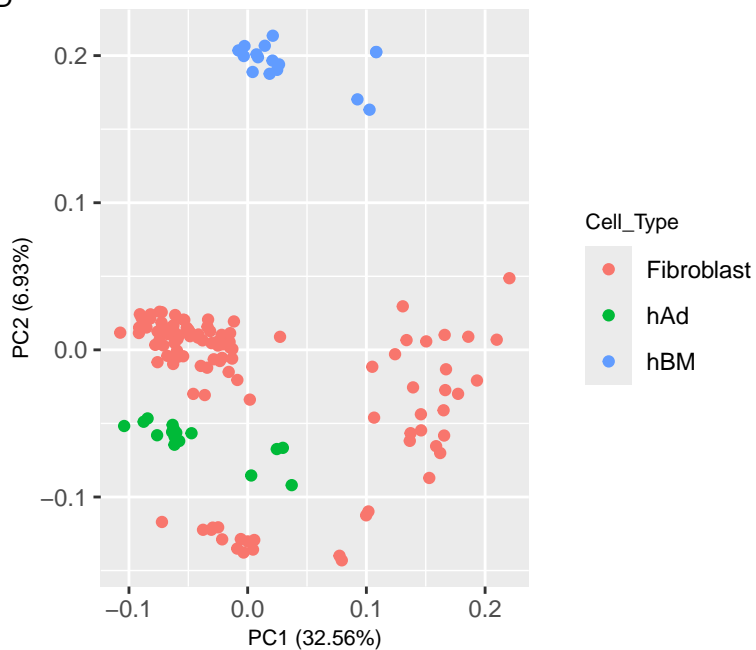

E

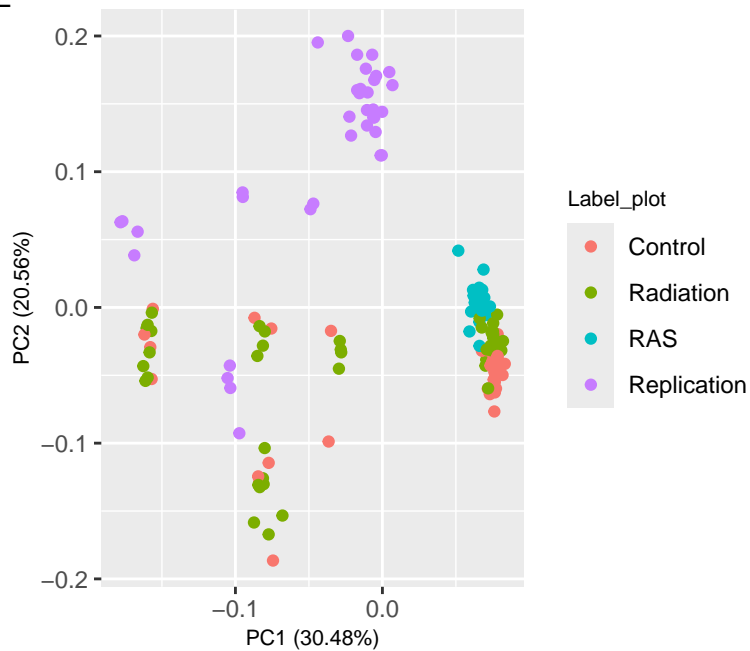

F

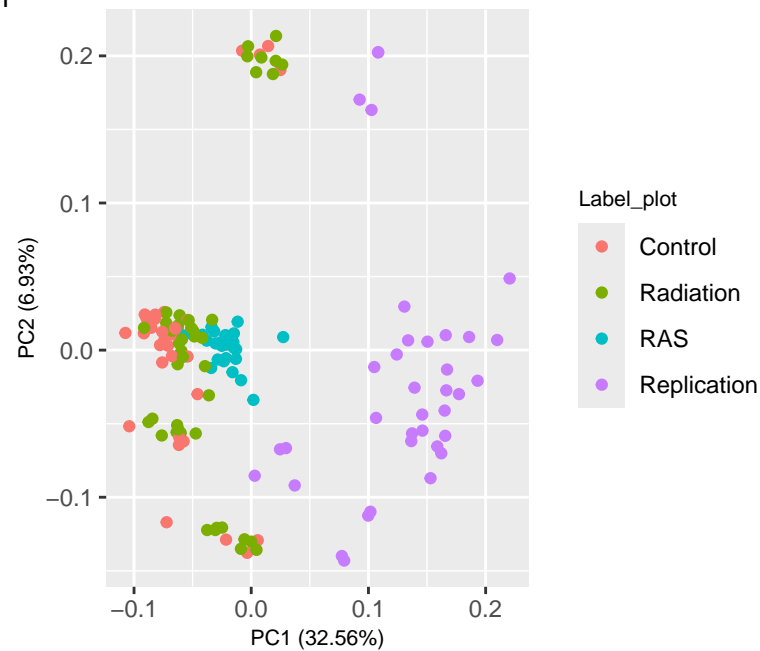

Supplement: Supplementary file 3 — Figure S3: Batch correction on GSE197723 and GSE227160 training datasets to remove technical variation before training our models. [file ACEL-25-e70430-s011.pdf]

# SenChronoAge- CpG Trait Enrichment Analysis

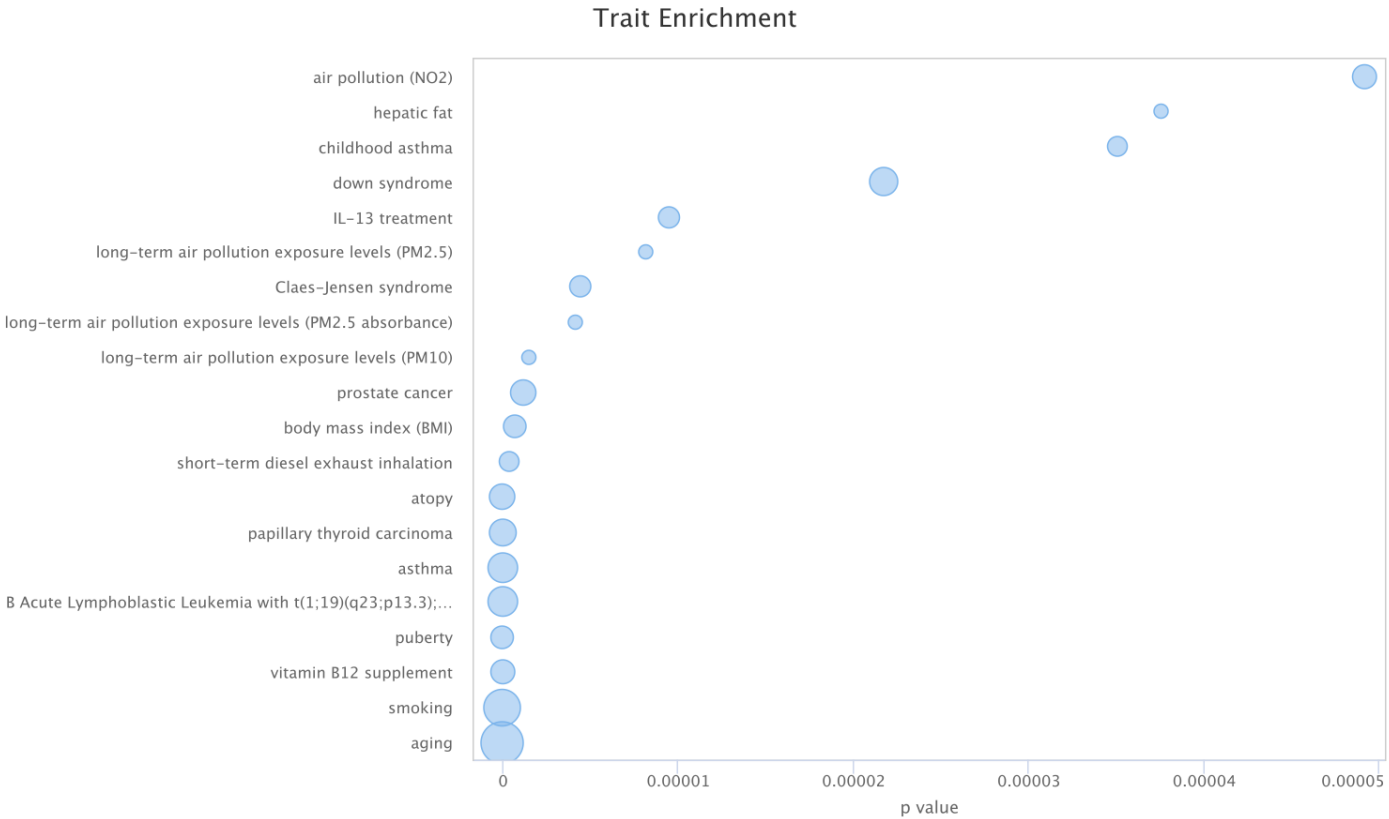

# SenChronoAge- GO and KEGG Terms

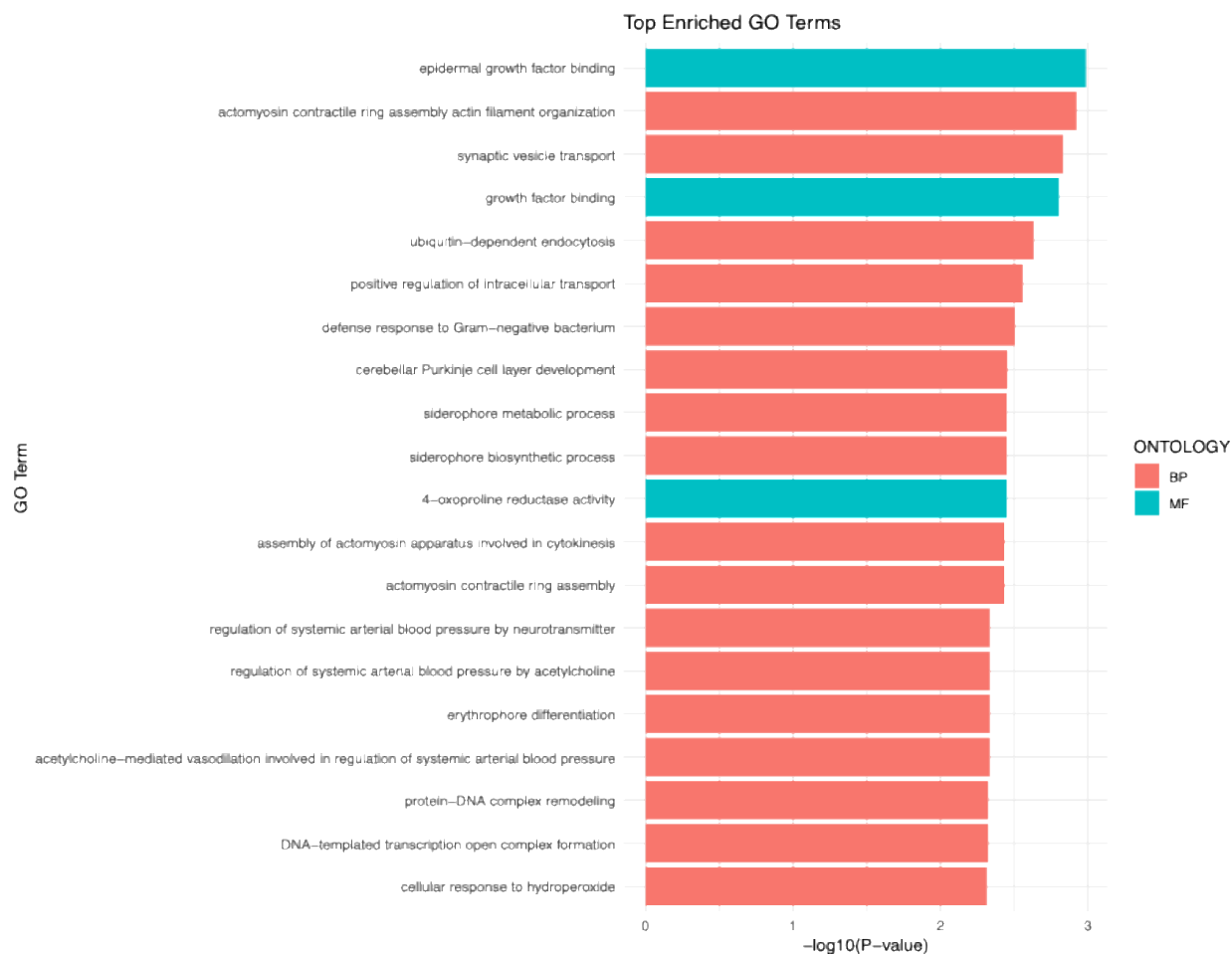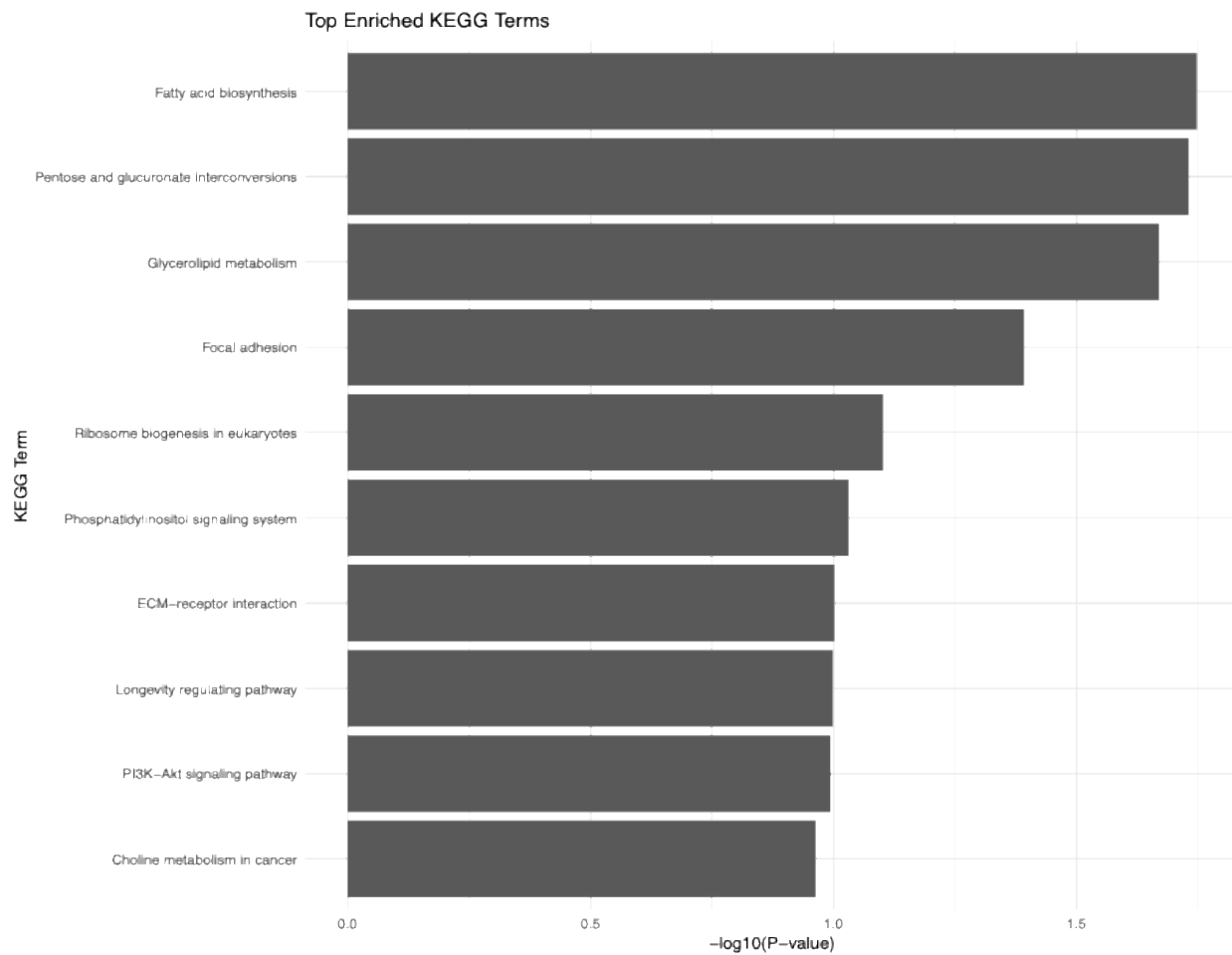

Supplement: Supplementary file 5 — Figure S5: Trait, GO, and KEGG enrichment analysis for 188 CpGs in SenChronoAge. [file ACEL-25-e70430-s008.pdf]

# SenMortalityAge- CpG Trait Enrichment Analysis

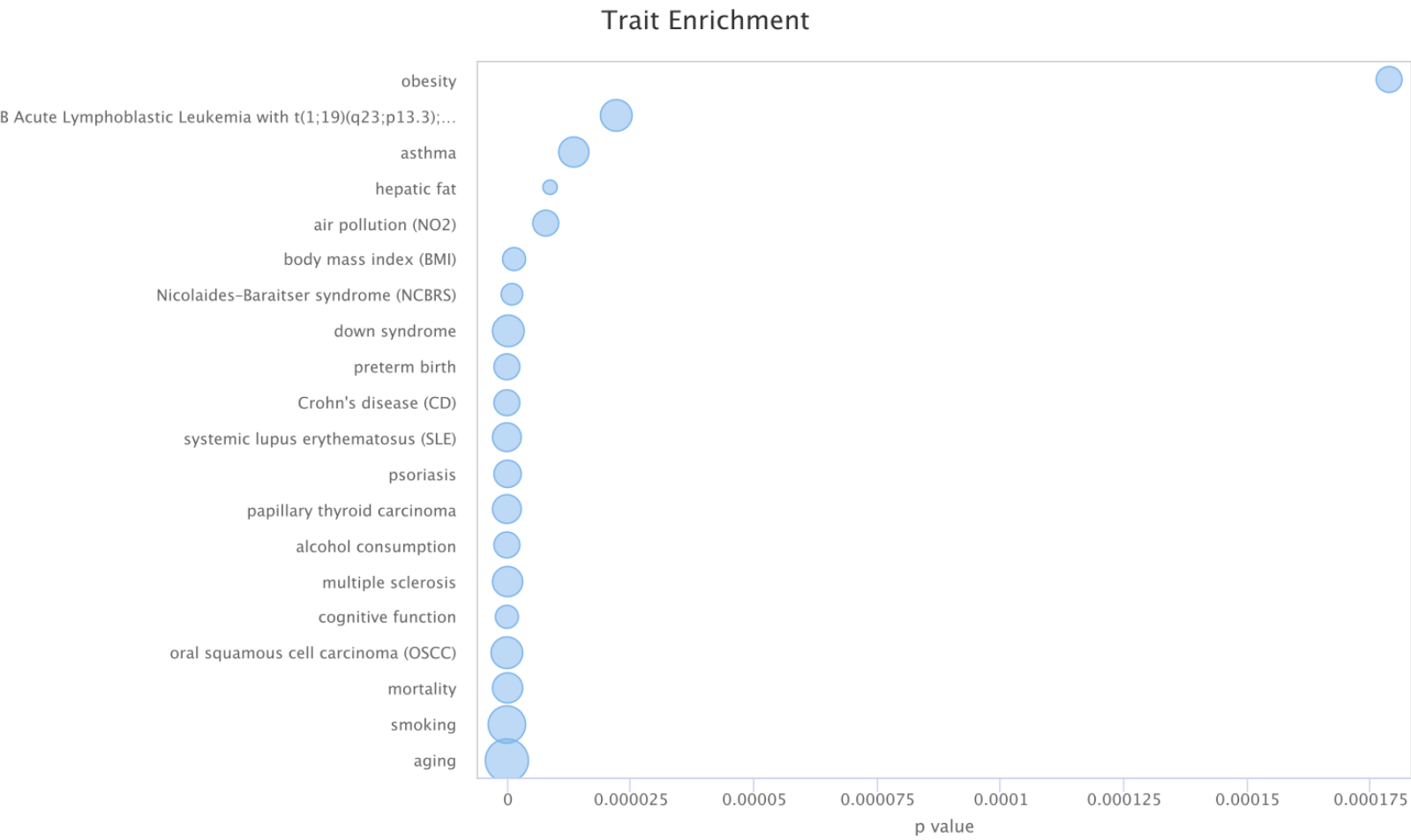

# SenMortalityAge- GO and KEGG Terms

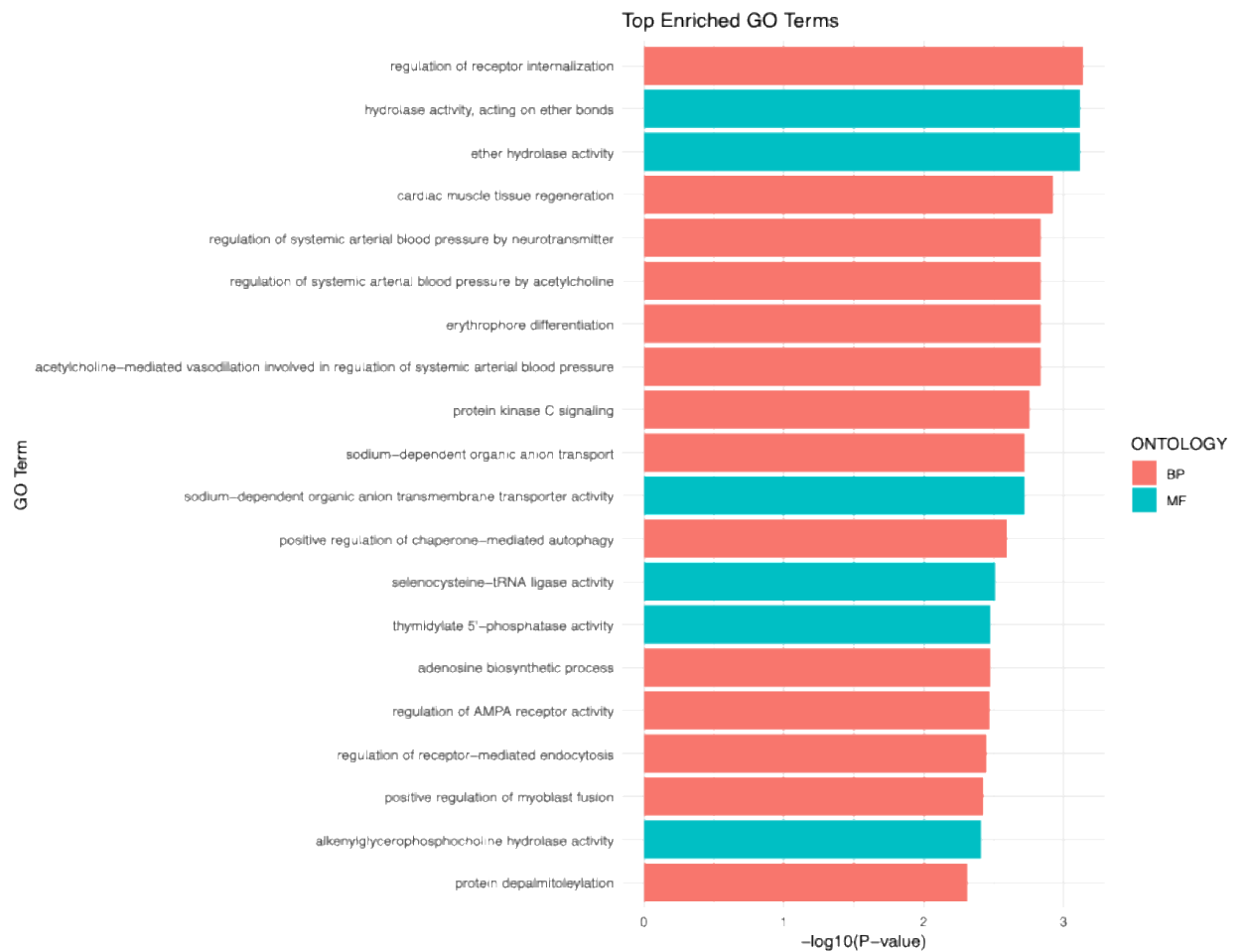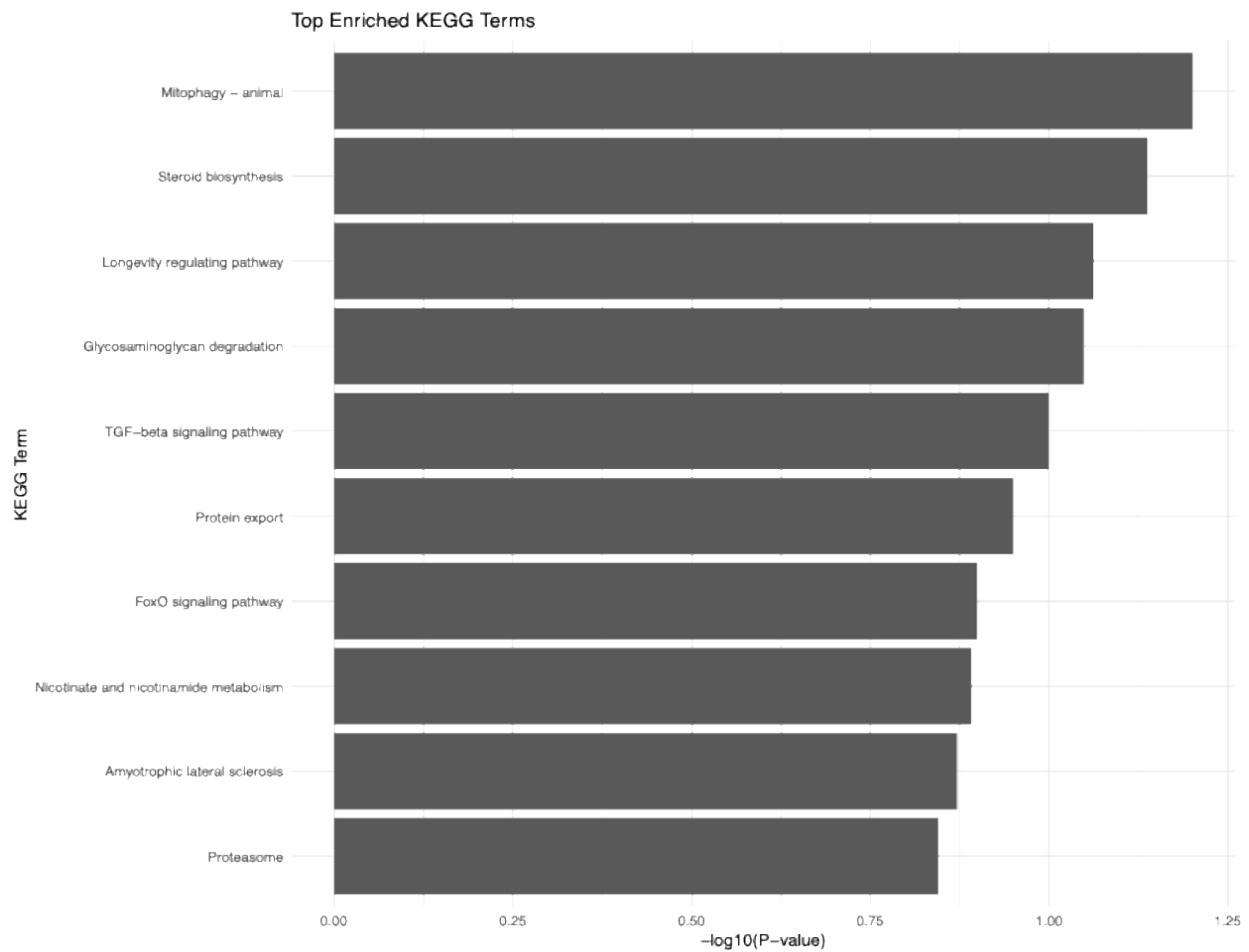

Supplement: Supplementary file 6 — Figure S6: Trait, GO, and KEGG enrichment analysis for 89 CpGs in SenMortalityAge. [file ACEL-25-e70430-s012.pdf]

A

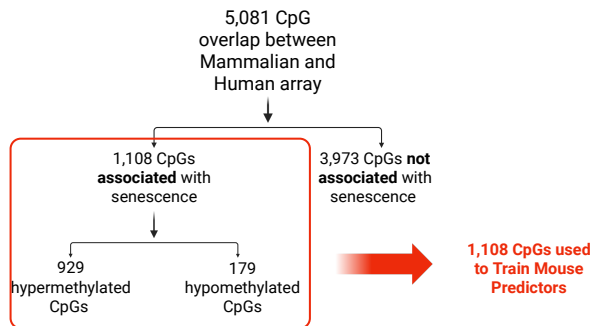

B

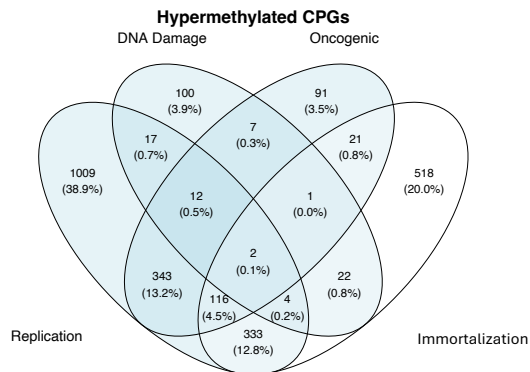

C

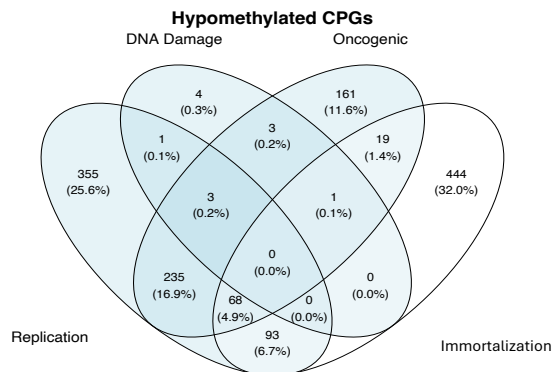

Supplement: Supplementary file 7 — Figure S7: (A) Pipeline for selecting CpGs for our mouse senescence model. Figure 7B and C. Venn diagram illustrating overlaps between senescence‐related and immortalization‐related CpGs for hypermethylation (B) and hypomethylation (C) in mouse‐senescence models. [file ACEL-25-e70430-s007.pdf]

# Sensitivity Analysis- Replicative Senescence

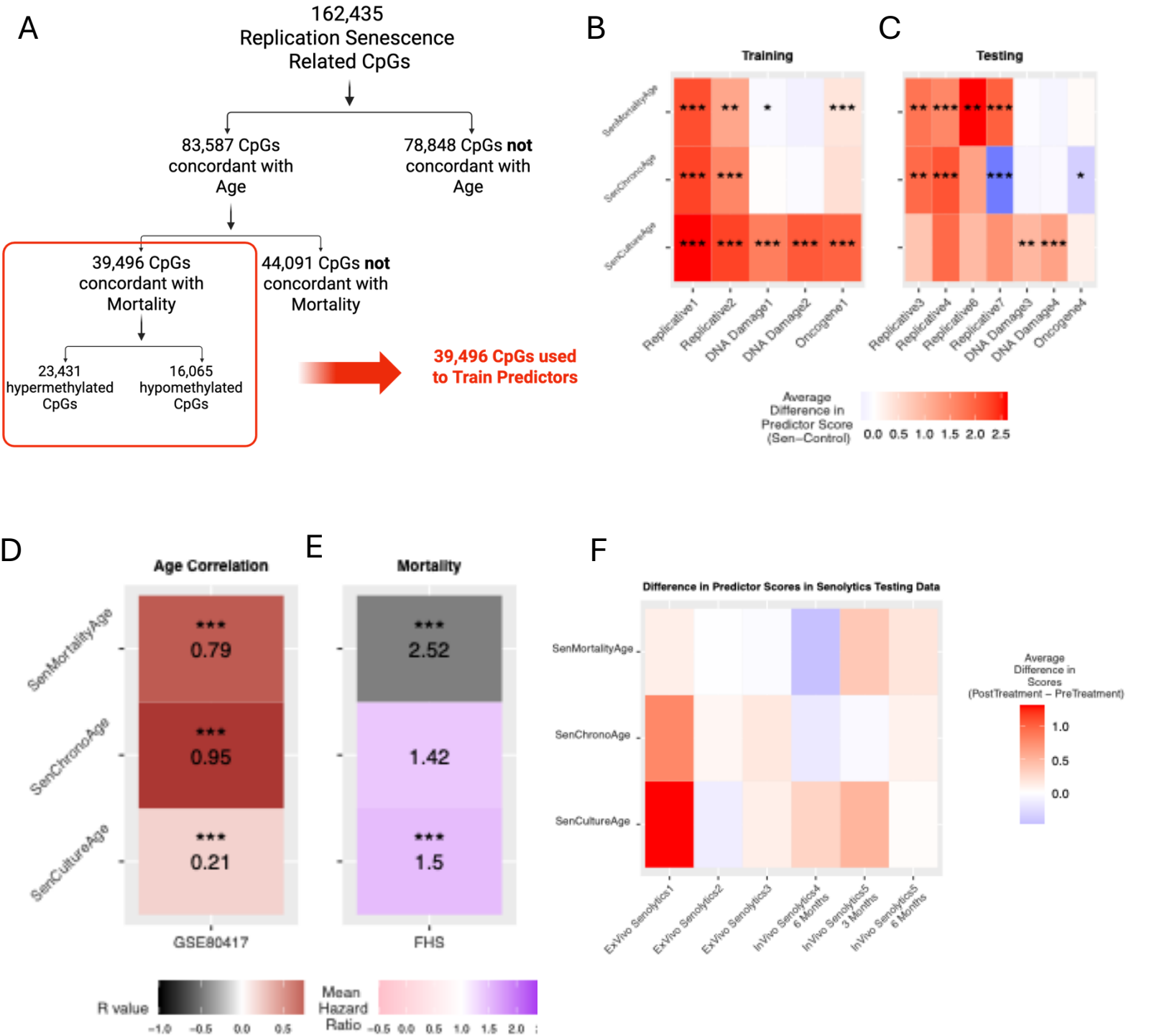

Supplement: Supplementary file 11 — Figure S11: Sensitivity Analysis for Replicative senescence. (A) Pipeline for selecting replication senescence CpGs for sensitivity analysis. Figure S11B,C Heatmap displaying differences between senescent and control samples in training datasets (B) and testing datasets (C) for 3 models (SenCultureAge, SenChronoAge, and SenMortalityAge). Figure S11D. Heatmap displaying age correlation coefficient (r) in testing dataset for each predictor. Figure S11E. Heatmap displaying standardized hazard ratio in testing split of FHS dataset for each predictor. Figure S11F Heatmap illustrating the mean difference of all three predictors in post‐senolytic treatment samples and pre‐senolytic treatment samples. [file ACEL-25-e70430-s003.pdf]

Sensitivity Analysis- Oncogenic Senescence

A

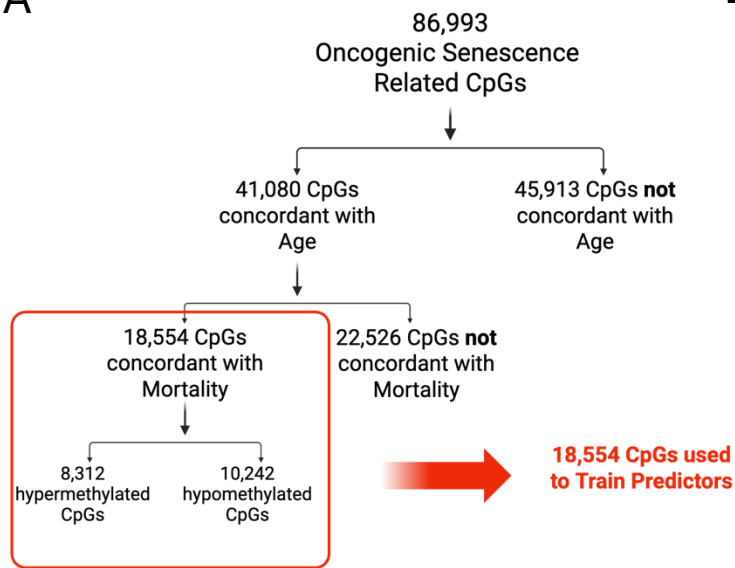

B

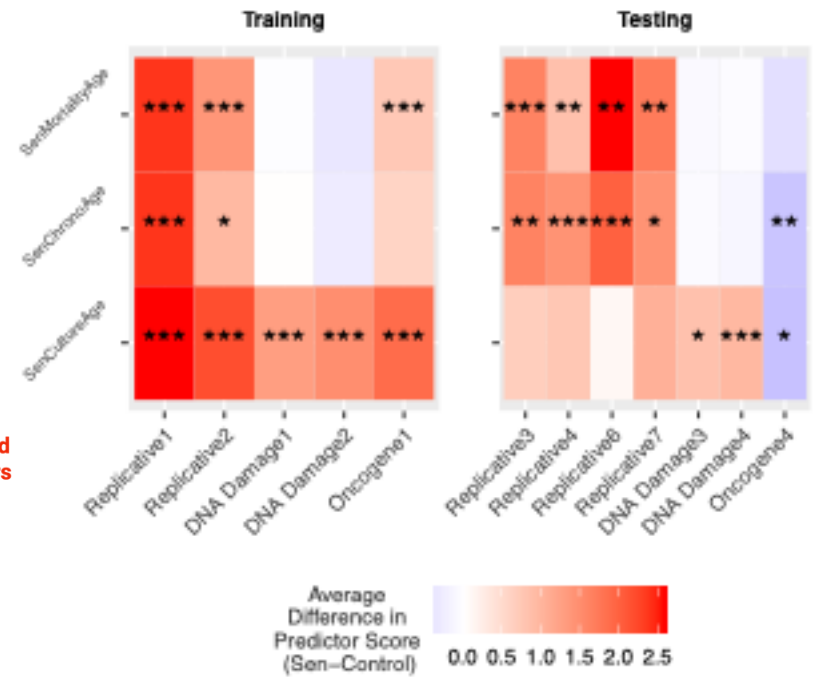

D

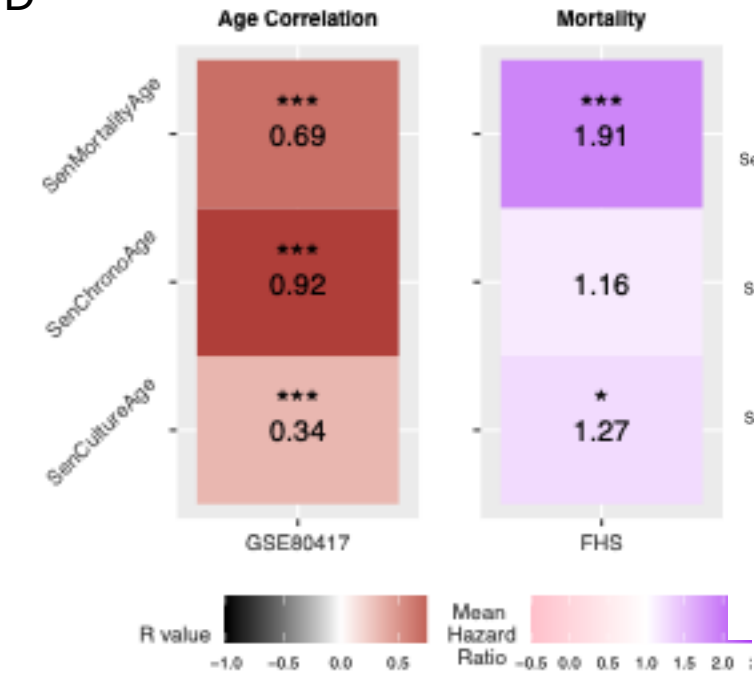

F

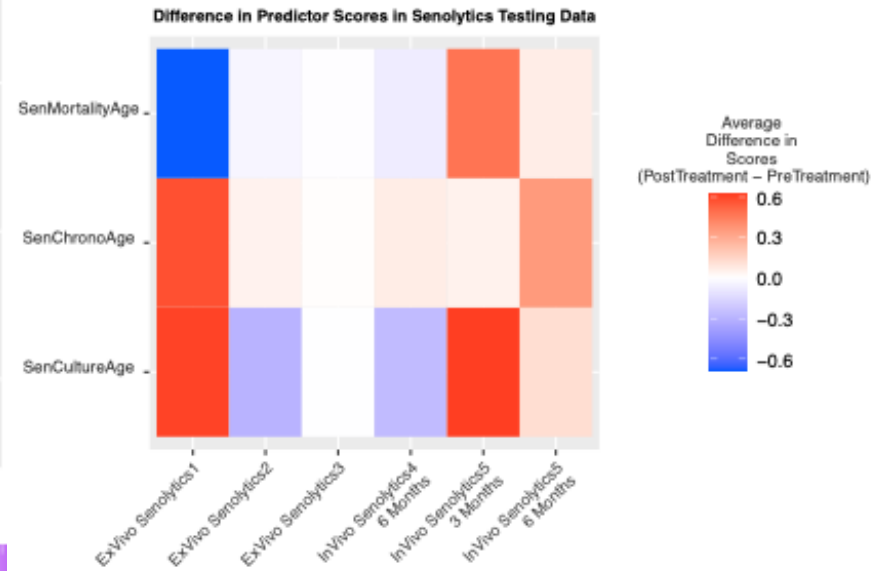

Supplement: Supplementary file 13 — Figure S13: Sensitivity analysis for Oncogenic senescence. Pipeline for selecting CpGs (A), predictor results in training (B) and testing (C) in vitro senescence, age correlation (D), hazard ratio (E), and senolytics validation (F). [file ACEL-25-e70430-s014.pdf]
