# Supplementary material for: DNA Methylation Signatures of Cellular Senescence Are Not Reversed by Senolytic Treatment
Source: Aging Cell. 2026 Feb 26;25(3):e70430. doi: 10.1111/acel.70430 (PMC12938503; doi:10.1111/acel.70430)

SenCultureAge- CpG Trait Enrichment Analysis

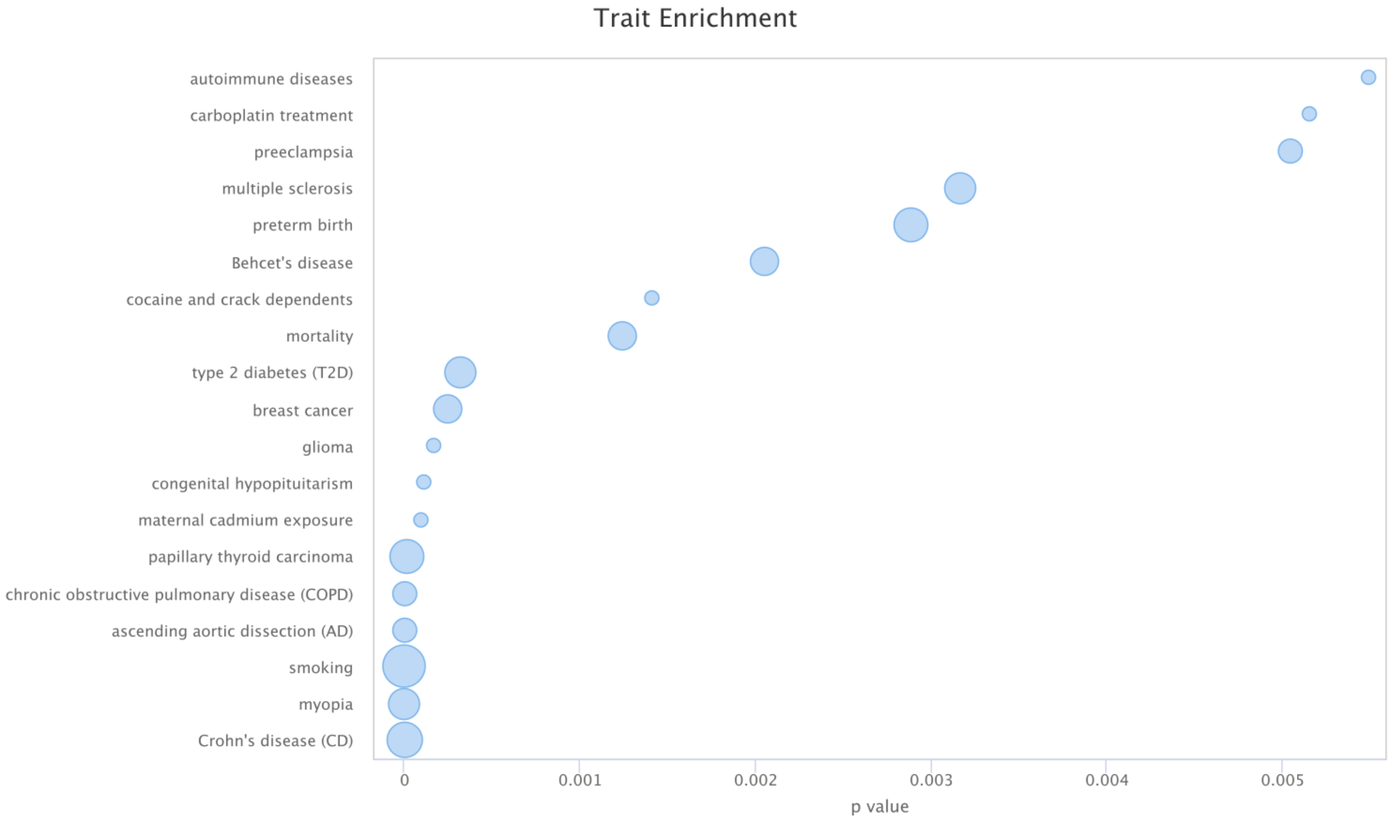

SenCultureAge- GO and KEGG Pathway Analyses

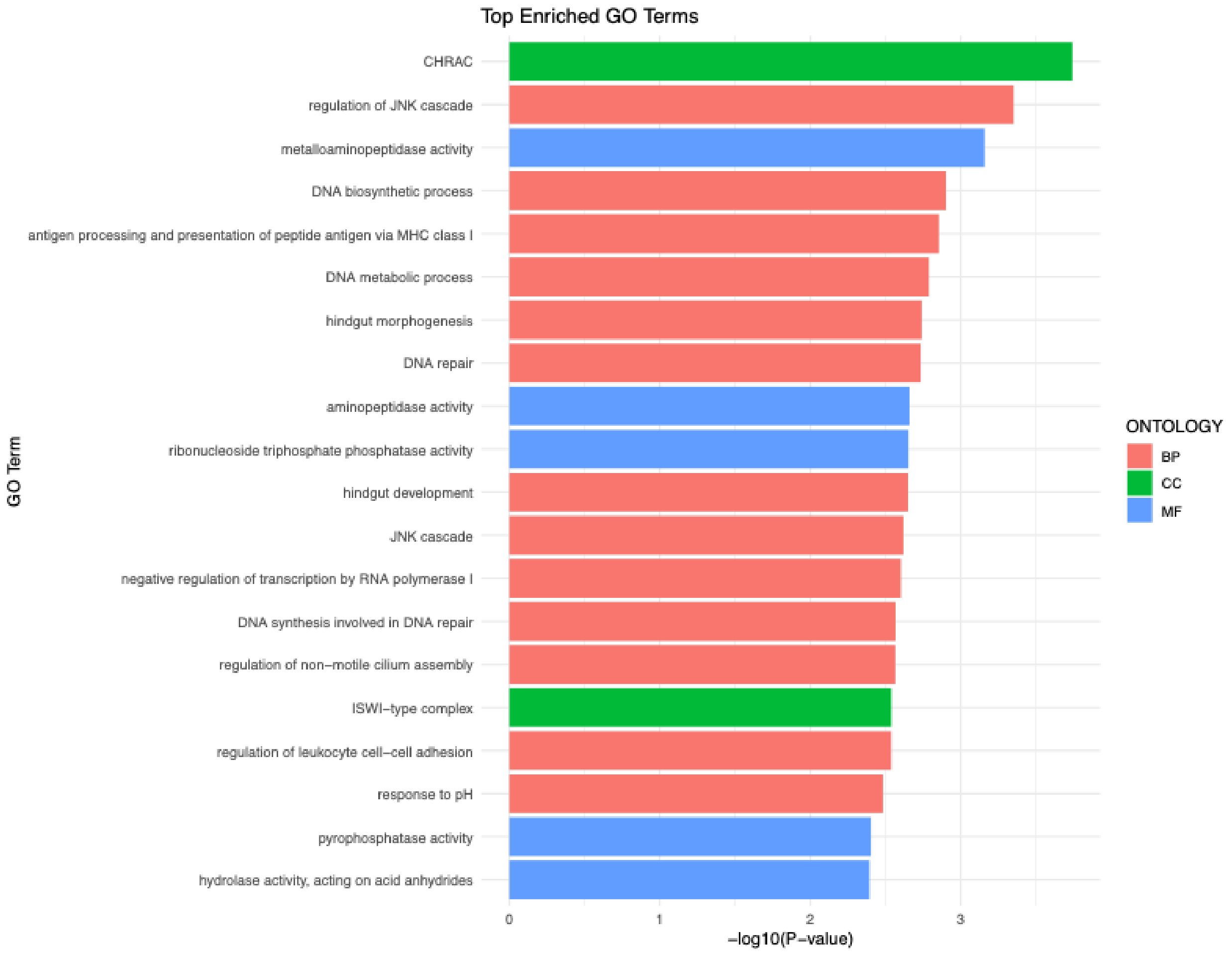

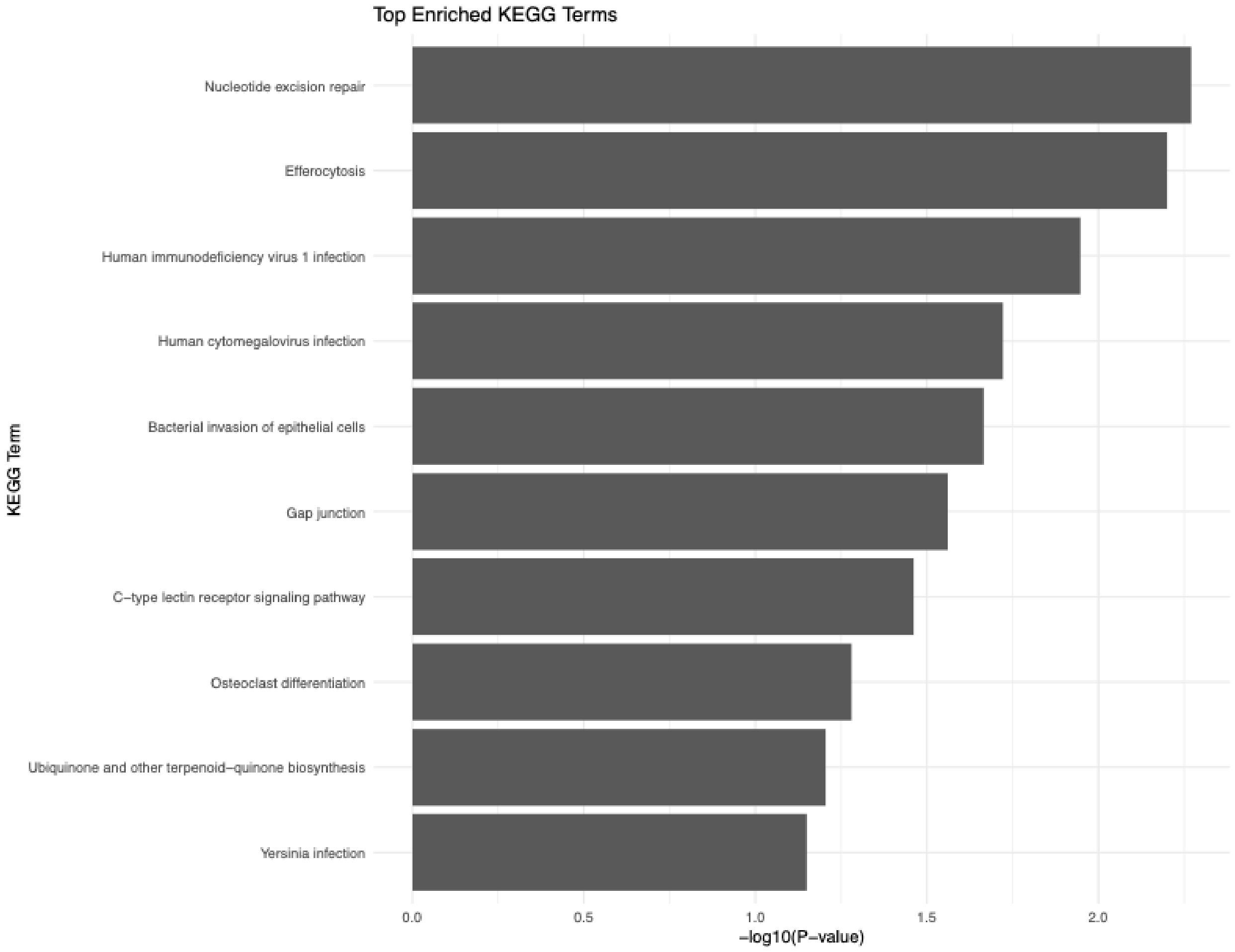

Supplement: Supplementary file 4 — Figure S4: Trait, GO, and KEGG enrichment analysis for 141 CpGs in SenCultureAge. [file ACEL-25-e70430-s005.pdf]
