# Supplementary material for: DNA Methylation Signatures of Cellular Senescence Are Not Reversed by Senolytic Treatment
Source: Aging Cell. 2026 Feb 26;25(3):e70430. doi: 10.1111/acel.70430 (PMC12938503; doi:10.1111/acel.70430)

SenMortalAge_Mouse Predictor

Applying predictor in Chambers et al. dataset to see how epigenetic age changes with:
1) control vs. injury mice in old and young mice
2) old vs. young mice in control and injured mice
3) No senolytic treatment vs. senolytic treatment in old control and old injured mice

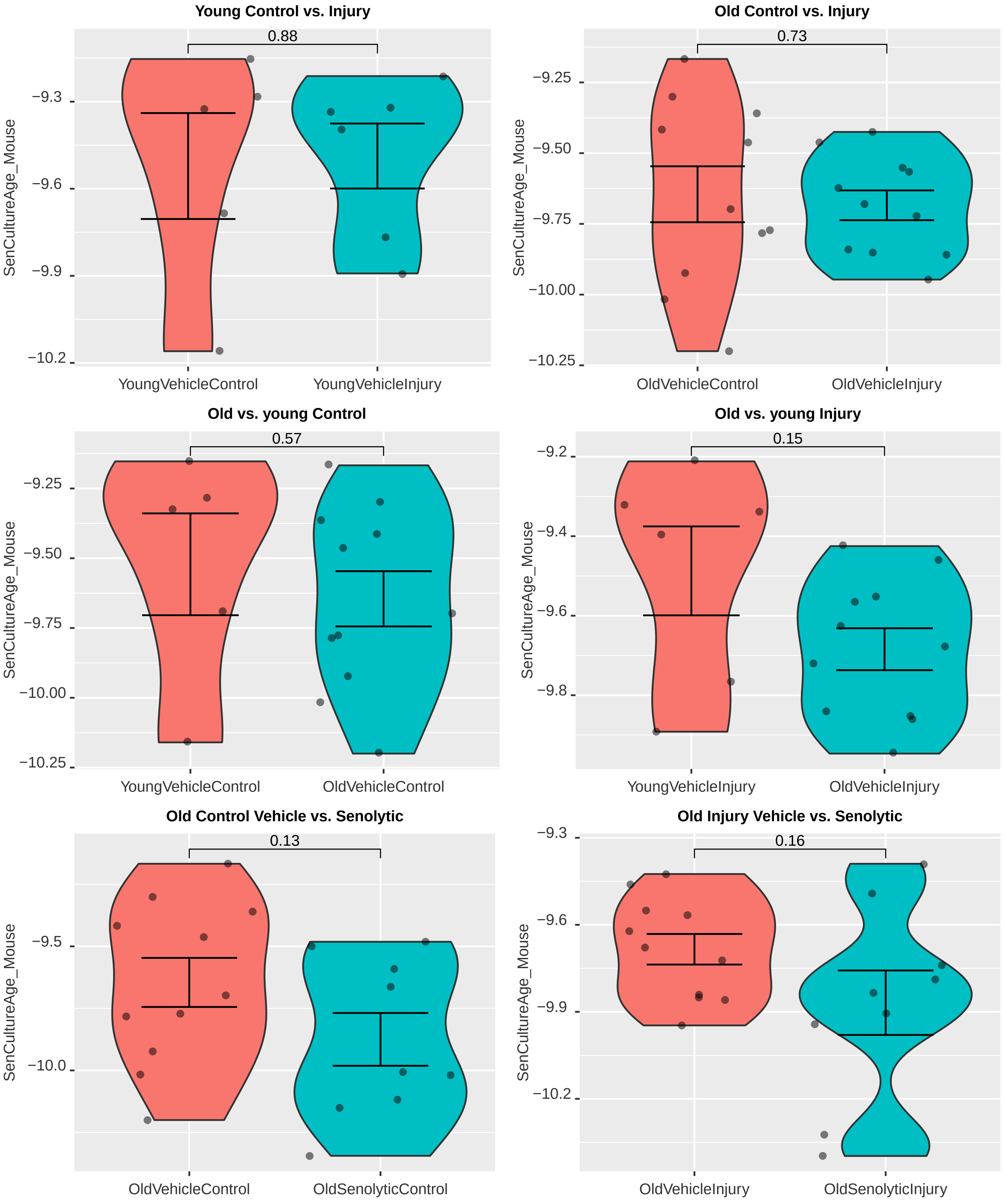

Supplement: Supplementary file 10 — Figure S10: SenMortalityAgeMouse predictor in Chambers et al. (2025) dataset, displaying change in predictor in control versus injury mice in old and young mice, old versus young mice in control and injured mice, and no senolytic treatment versus senolytic treatment in old control and old injured mice. [file ACEL-25-e70430-s004.pdf]
