# Supplementary material for: DNA Methylation Signatures of Cellular Senescence Are Not Reversed by Senolytic Treatment
Source: Aging Cell. 2026 Feb 26;25(3):e70430. doi: 10.1111/acel.70430 (PMC12938503; doi:10.1111/acel.70430)
Supplement: Supplementary file 12 — Figure S12: Sensitivity analysis for DNA damage senescence. Pipeline for selecting CpGs (A), predictor results in training (B) and testing (C) in vitro senescence, age correlation (D), hazard ratio (E), and senolytics validation (F). [file ACEL-25-e70430-s002.pdf]

Sensitivity Analysis- DNA Damage Senescence

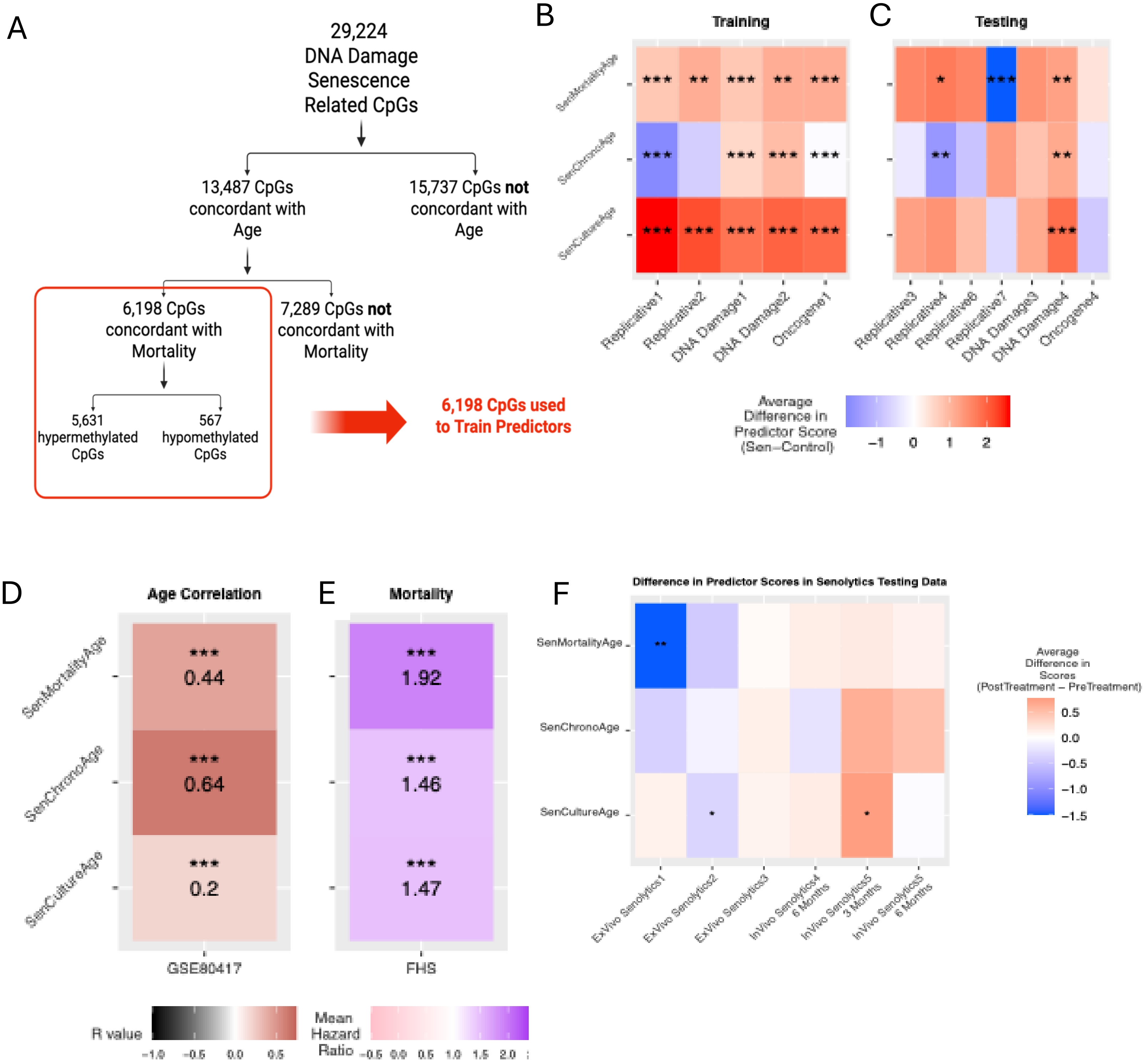

F

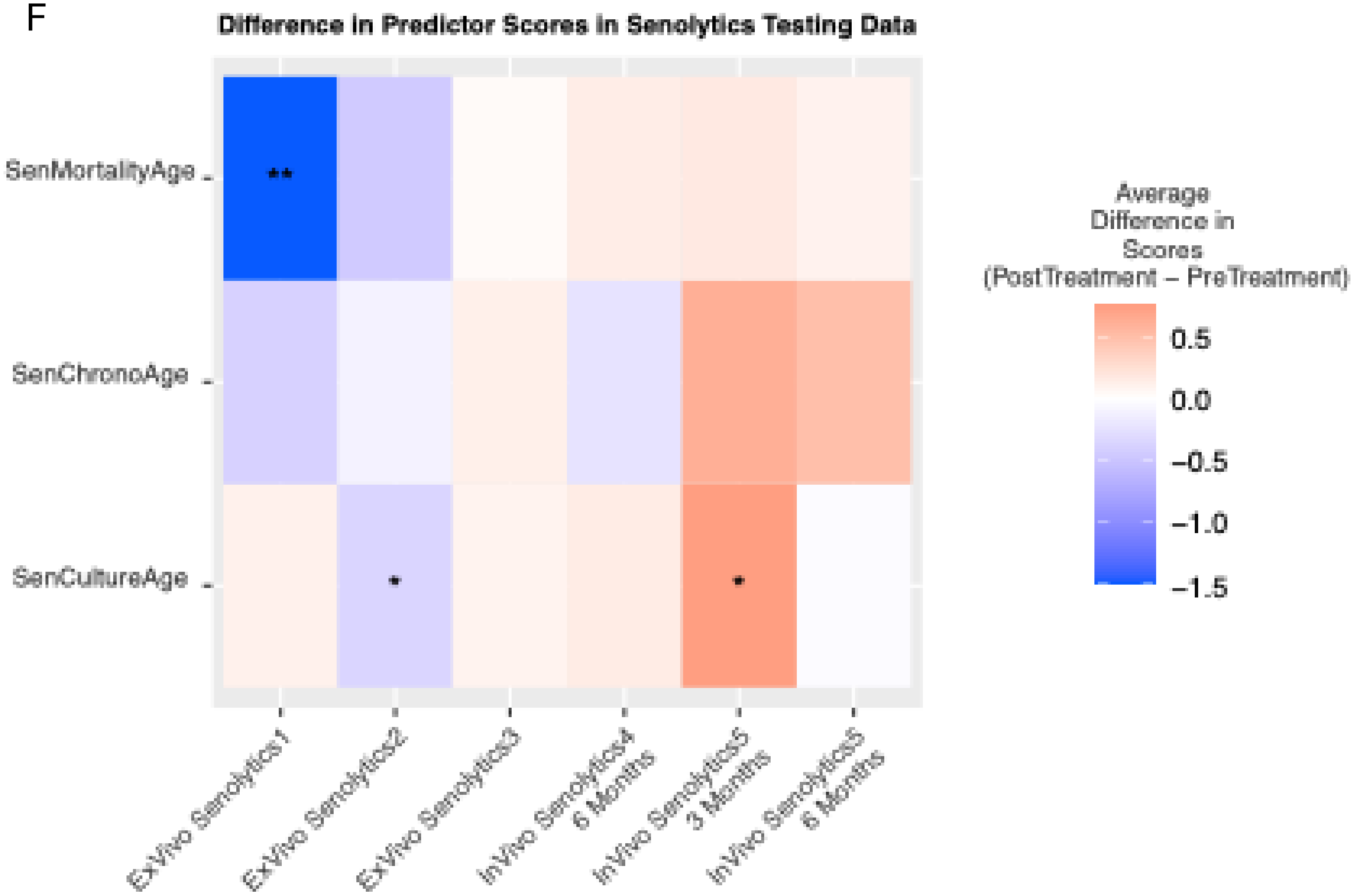
Difference in Predictor Scores in Senolytics Testing Data
SenMortalityAge
SenChronoAge
SenCultureAge
**
*
*
ExVivo Senolytics1
ExVivo Senolytics2
ExVivo Senolytics3
InVivo Senolytics4 6 Months
InVivo Senolytics5 3 Months
InVivo Senolytics5 6 Months
Average Difference in Scores (PostTreatment – PreTreatment)
0.5
0.0
−0.5
−1.0
−1.5
